# Uropathogenic *Escherichia coli* employs both evasion and resistance to subvert innate immune-mediated zinc toxicity for dissemination

**DOI:** 10.1073/pnas.1820870116

**Published:** 2019-03-07

**Authors:** Claudia J. Stocks, Minh-Duy Phan, Maud E. S. Achard, Nguyen Thi Khanh Nhu, Nicholas D. Condon, Jayde A. Gawthorne, Alvin W. Lo, Kate M. Peters, Alastair G. McEwan, Ronan Kapetanovic, Mark A. Schembri, Matthew J. Sweet

**Affiliations:** ^a^Institute for Molecular Bioscience, University of Queensland, St. Lucia, QLD 4072, Australia;; ^b^Institute for Molecular Bioscience Centre for Inflammation and Disease Research, University of Queensland, St. Lucia, QLD 4072, Australia;; ^c^Australian Infectious Diseases Research Centre, University of Queensland, St. Lucia, QLD 4072, Australia;; ^d^School of Chemistry and Molecular Biosciences, University of Queensland, St. Lucia, QLD 4072, Australia

**Keywords:** antimicrobial responses, macrophage, TraDIS, UPEC, zinc

## Abstract

Toll-like receptor (TLR)-inducible zinc toxicity is a recently described macrophage antimicrobial response used against bacterial pathogens. Here we investigated deployment of this pathway against uropathogenic *Escherichia coli* (UPEC), the major cause of urinary tract infections. Primary human macrophages subjected EC958, a representative strain of the globally disseminated multidrug-resistant UPEC ST131 clone, to zinc stress. We therefore used transposon-directed insertion site sequencing to identify the complete set of UPEC genes conferring protection against zinc toxicity. Surprisingly, zinc-susceptible EC958 mutants were not compromised for intramacrophage survival, whereas corresponding mutants in the nonpathogenic *E. coli* K-12 strain MG1655 displayed significantly reduced intracellular bacterial loads within human macrophages. To investigate whether the intramacrophage zinc stress response of EC958 reflected the response of only a subpopulation of bacteria, we generated and validated reporter systems as highly specific sensors of zinc stress. Using these tools we show that, in contrast to MG1655, the majority of intramacrophage EC958 evades the zinc toxicity response, enabling survival within these cells. In addition, EC958 has a higher tolerance to zinc than MG1655, with this likely being important for survival of the minor subset of UPEC cells exposed to innate immune-mediated zinc stress. Indeed, analysis of zinc stress reporter strains and zinc-sensitive mutants in an intraperitoneal challenge model in mice revealed that EC958 employs both evasion and resistance against zinc toxicity, enabling its dissemination to the liver and spleen. We thus demonstrate that a pathogen of global significance uses multiple mechanisms to effectively subvert innate immune-mediated zinc poisoning for systemic spread.

## Significance

Uropathogenic *Escherichia coli* (UPEC) is responsible for most urinary tract infections and is also a frequent cause of sepsis, thus necessitating an understanding of UPEC-mediated subversion of innate immunity. The role of zinc in the innate immune response against UPEC infection, and whether this pathogen counters this response, has not been examined. Here we demonstrate, both in vitro and in vivo, that UPEC both evades and resists innate immune-mediated zinc toxicity to persist and disseminate within the host. Moreover, we have defined the set of UPEC genes conferring zinc resistance and have developed highly selective *E. coli* reporter systems to track zinc toxicity. These innovative approaches substantially enhance our understanding of immune-mediated metal ion toxicity and bacterial pathogenesis.

Urinary tract infections (UTIs) are one of the most common infections worldwide, affecting nearly half of all women at least once in their lifetime (1). UTIs primarily present as acute bladder infections, but can lead to dangerous sequelae, including pyelonephritis and septicaemia. With a global disease burden of ∼150 million cases, the emergence of multidrug resistance in UTI-causing pathogens is a major public health concern (2). Uropathogenic *Escherichia coli* (UPEC) is the causative agent of the majority of UTIs, and is increasingly associated with resistance to multiple antibiotics from different classes, as exemplified by the globally disseminated ST131 clone (3). The multifaceted intra- and extracellular lifestyles of UPEC, including its ability to form intracellular bacterial communities protected from inflammatory cells, host-generated antimicrobial molecules, and antibiotics (4, 5), necessitates the discovery of new strategies to combat UPEC infections.

After breaching epithelial cell barriers, invading UPEC encounter macrophages and neutrophils, phagocytic cells that actively take up and destroy foreign material. While neutrophils play a critical role in UPEC clearance (6), the role of macrophages in UPEC infection is complex and less well understood. A bladder-resident macrophage population has an essential role in mediating neutrophil recruitment to the infected bladder (7), but some UPEC strains can attenuate neutrophil function and migration (8). Certain strains, such as CFT073, can trigger rapid macrophage cell death (9, 10), which may be one mechanism that disables neutrophil recruitment. Studies with *Cd14*^−/−^ mice and the macrophage-depleting agent clodronate further highlight an essential role for these cells in host defense against UPEC (11). However, other UPEC strains can actually survive within macrophages (12), thus highlighting the complexity in macrophage–UPEC dynamics. The interactions between ST131 strains and macrophages have not been extensively studied.

Professional intramacrophage pathogens, such as *Salmonella enterica* serovar Typhimurium, must evade a battery of macrophage antimicrobial responses, such as inducible production of reactive oxygen and nitrogen species. Some of these are Toll-like receptor (TLR)-inducible late-stage responses designed to counteract pathogens that persist within the intramacrophage environment (13). One such response involves the manipulation of key metal ions. In this regard, restriction of essential metal ion availability and direct metal ion toxicity have both been documented during intramacrophage infection (14, 15). For example, human macrophages subject *Mycobacterium tuberculosis* to a zinc toxicity response (16), a response that *S*. Typhimurium evades via a mechanism dependent on *Salmonella* pathogenicity island-1 (SPI-1) (17). Harnessing of zinc toxicity may be a conserved innate immune antimicrobial response, with neutrophils mobilizing high levels of zinc toward engulfed *Streptococcus* (18, 19).

Excess zinc can be toxic to bacteria through multiple mechanisms, including enhanced susceptibility to oxidative stress triggered by induced deficiency in manganese (20). Zinc stress also inhibits key glycolytic enzymes, impairing glucose metabolism and capsule biosynthesis (21), and displacing iron in iron–sulfur complexes of metabolic enzymes (22). In this context, elevated zinc is predicted to exert a direct antibacterial effect within macrophages. Our limited understanding of the mechanisms involved in this innate immune antimicrobial response partly stems from a lack of reliable tools to monitor it. In this study, we developed zinc-stress reporter strains and used a transposon-directed insertion site sequencing (TraDIS) screen in EC958, a representative strain of the ST131 clone (23), to investigate deployment of innate immune-mediated zinc stress against UPEC. Our findings highlight the importance of macrophage-mediated zinc toxicity in host defense, and reveal that EC958 effectively subverts this response through both evasion and resistance, thus enabling dissemination within the host.

## Results

### Human Macrophages Subject the UPEC Strain EC958 to a Zinc Stress Response.

**Fig. 1.**
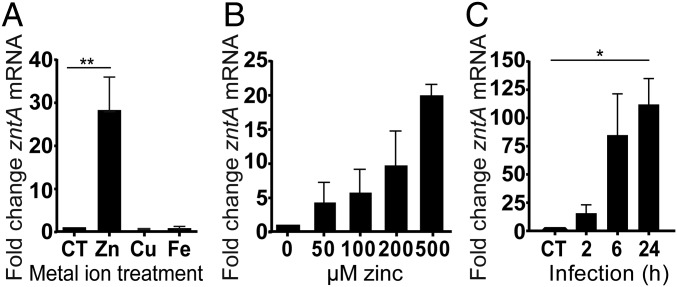
UPEC strain EC958 is exposed to zinc stress within the intramacrophage environment. (*A*) EC958 was incubated for 6 h in complete RPMI media containing 500 µM ZnSO_4_, CuSO_4_, FeSO_4_, or water (control, CT), after which *zntA* mRNA levels, relative to *gapA*, were determined by quantitative real-time PCR. Data (mean + SEM, *n* = 3), expressed as fold-change compared with the control, are combined from three experiments (**P < 0.01, one-way ANOVA and Dunnetts’s multiple comparisons test). (*B*) EC958 was incubated as above in increasing zinc concentrations. Levels of *zntA* mRNA (relative to *gapA*) were determined by quantitative real-time PCR. Data (mean + range, *n* = 2), expressed as fold-change compared with the control, are combined from two experiments. (*C*) HMDM were infected with EC958 (MOI of 100) for 1 h, whereupon extracellular bacteria were removed by gentamicin exclusion. Cells were lysed at 2, 6, or 24 hpi, after which RNA was extracted. Levels of *zntA* mRNA, relative to *gapA*, were determined by quantitative real-time PCR. Bacteria were cultured for 2 h in complete RPMI media alone as a control. Data (mean + SEM, *n* = 3), expressed as fold-change compared with the control, are combined from three experiments (*P < 0.05, one-way ANOVA and Dunnett's multiple comparisons test).

We began by determining whether there was any evidence that macrophages can subject UPEC to zinc stress. To do so, we focused on EC958, as a representative fluoroquinolone-resistant strain of the globally disseminated ST131 clone. *E. coli* and *S*. Typhimurium up-regulate expression of the transporter ZntA to efflux zinc when cytotoxic levels of this metal ion are encountered (24, 25); however, copper also up-regulates *zntA* mRNA in *S*. Typhimurium (17). Moreover, we (26, 27) and others (28, 29) have shown that copper has an important role in innate immune antimicrobial responses against Gram-negative bacteria. Therefore, we first investigated the specificity of zinc-inducible *zntA* expression in EC958. In this *E. coli* strain, *zntA* was induced by zinc, but not copper or iron (Fig. 1*A*). Analysis of the growth and survival of EC958 in complete RPMI containing 500 µM CuSO_4_ (*SI Appendix*, Fig. S1*A*) demonstrated that the failure of copper to induce *zntA* was not caused by copper toxicity. We note that the concentration of copper that could be tolerated by EC958 was significantly higher than previously reported for a K-12 strain grown in M9 minimal medium supplemented with glucose (30). Because 500 µM CuSO_4_ also did not affect the growth of either EC958 or *E. coli* K-12 strain MG1655 in Lysogeny broth (LB) (*SI Appendix*, Fig. S1*B*), we attribute this difference in copper sensitivity to the different media used (complete RPMI or LB versus M9 minimal). The response to zinc occurred in a concentration-dependent manner, with the lowest concentration examined (50 µM), causing an ∼fourfold increase in *zntA* mRNA levels (Fig. 1*B*). We thus used *zntA* expression as a biologically relevant readout of zinc stress in infected macrophages. Between 2 and 6 h postinfection (hpi) of primary human monocyte-derived macrophages (HMDM), EC958 strongly up-regulated *zntA* transcription, indicating zinc stress, with expression remaining elevated at 24 hpi (Fig. 1*C* and *SI Appendix*, Fig. S1 *C* and *D*).

### TraDIS Identifies the Set of EC958 Genes That Mediate Zinc Resistance.

**Fig. 2.**
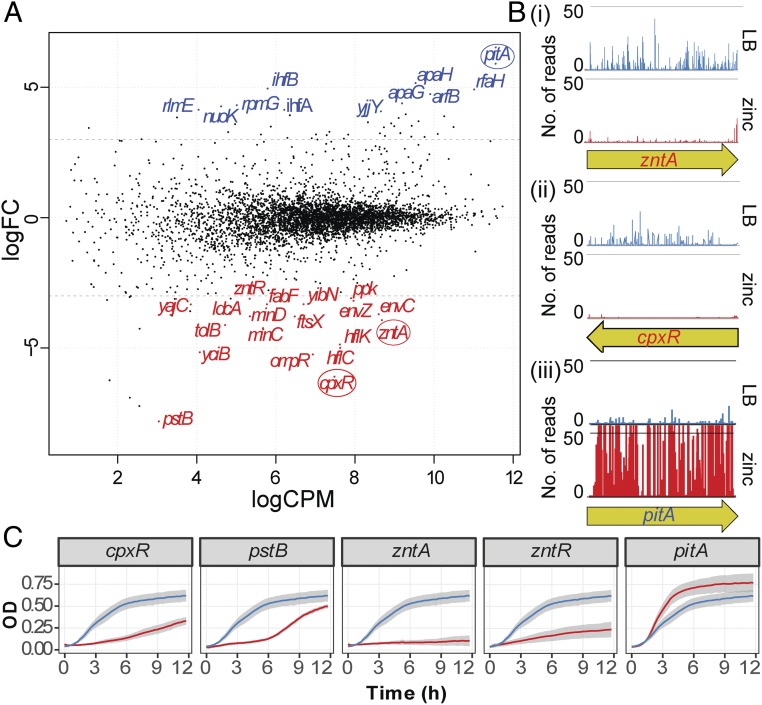
Identification and characterization of UPEC genes that influence growth in the presence of zinc. (*A*) Identification by TraDIS analysis of EC958 genes that regulate growth in 1 mM ZnSO_4._ Genes required for growth in zinc [logFC < −3 and false-discovery rate (FDR) < 0.001] are shown in red. Genes that, when mutated, promote growth in zinc (logFC > 4 and FDR < 0.001) are shown in blue. (*B*) Examples of reads mapped to individual insertion sites and their differences between control (LB) and test (LB-Zn) conditions for the *zntA* (*i*), *cpxR* (*ii*), and *pitA* (*iii*) genes. (*C*) Growth kinetics of wild-type (blue line) versus mutants (red line: ∆*cpxR*, ∆*pstB*, ∆*zntA*, ∆*zntR*, and ∆*pitA*) in LB-Zn conditions. Data (*n* = 3) are combined from three experiments, presented as mean (solid line) ± SEM (gray shades).

Because our initial experiments suggested that human macrophages subject EC958 to zinc stress, we next performed a genetic screen to identify genes required for zinc resistance. We incubated ∼1 million EC958 transposon mutants in 1 mM ZnSO_4_ in LB broth for 18 h, then recovered surviving cells and isolated genomic DNA. We then performed TraDIS and data analysis, as previously described (31, 32), to identify: (*i*) genes whose mutation led to increased susceptibility to killing by zinc, and (*ii*) genes whose mutation led to increased tolerance to zinc. The former had a significant reduction in read counts (i.e., fewer transposon insertions) in the test samples vs. the control samples [log_2_ fold-change (logFC) < −3.0; *P* < 0.001] (Fig. 2*A*, red), whereas the latter had a significant increase in read counts (i.e., more transposon insertions) (logFC > 4.0; *P* < 0.001) (Fig. 2*A*, blue).

This screen identified *zntA*, as well as *zntR*, which encodes a zinc-dependent activator of *zntA*; these genes are known to be required for zinc-resistance (24, 33), confirming the validity of our screen. In addition, other genes whose mutation led to increased susceptibility to zinc included those involved in response to stress (*cpxR*, *envC*, *envZ*, *ompR*), phosphate transport (*pstB*), peptidoglycan synthesis (*ldcA*, *yciB*), and outer membrane stability (*tolB*) (Fig. 2*A* and *SI Appendix*, Table S1). Despite their diversity in functions, many of these genes share commonality in that they are linked to stress responses associated with perturbation of membrane integrity. Conversely, mutation of *pitA*, which encodes a low-affinity zinc transporter, resulted in increased tolerance to zinc (Fig. 2*A* and *SI Appendix*, Table S1). This is consistent with a previous analysis of this gene in *E. coli* K-12 (34). The read-count data of *zntA* and *cpxR*, as well as *pitA*, are displayed in Fig. 2*B*. We next generated defined mutants in EC958 corresponding to all 30 genes identified in the TraDIS screen, and confirmed the phenotype for 22 (73%) of these mutants (Fig. 2*C* and *SI Appendix*, Fig. S2 and Table S1). Of the mutants validated for altered zinc sensitivity, 17 were confirmed through comparison of growth kinetics for wild-type versus mutant strains in 1 mM ZnSO_4_, while another 5 had more subtle phenotypes only revealed in competitive growth assays with the wild-type strain in 1 mM ZnSO_4_. Fig. 2*C* shows that the *cpxR*, *pstB, zntA*, and *zntR* EC958 mutants all display increased zinc sensitivity, whereas the converse is true for the *pitA* mutant. Given that many of the genes that confer zinc tolerance in EC958 (Fig. 2 and *SI Appendix*, Table S1) are linked to cell envelope integrity, we next assessed whether innate immune antimicrobial mechanisms that permeabilize and damage cell membranes influence zinc susceptibility. The macrophage-expressed antimicrobial peptide cathelicidin (35, 36) did not influence zinc toxicity against either wild-type EC958 or an EC958 *zntA* mutant (*SI Appendix*, Table S2), but a contrasting effect was observed for reactive oxygen species. The superoxide anion generator paraquat increased zinc sensitivity of the EC958 *zntA* mutant, compared with wild-type EC958 (*SI Appendix*, Fig. S3). Collectively, these findings confirm that our approach has mapped the zinc resistome of a clinically relevant UPEC strain, and support the view that zinc toxicity is relevant to mechanisms employed by the innate immune system for bacterial clearance.

### EC958 Zinc Susceptibility Genes Are Not Required for Intramacrophage Survival.

**Fig. 3.**
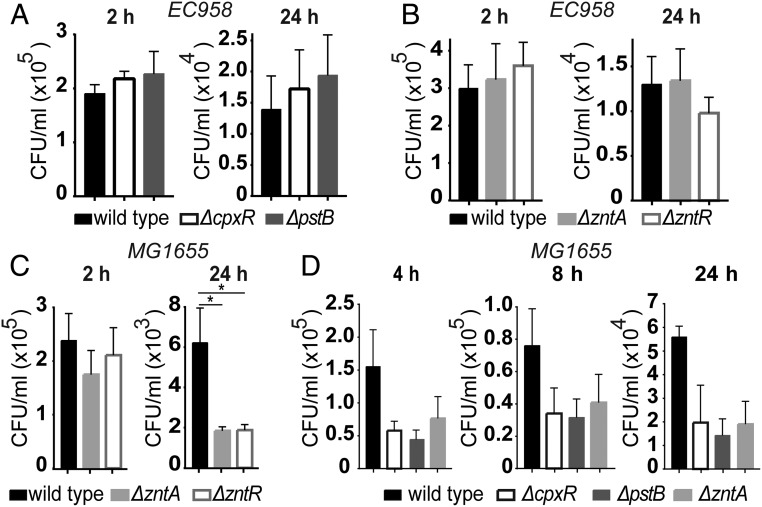
EC958 evades the zinc toxicity response of human macrophages. (*A* and *B*) HMDM were infected with wild-type EC958 or the indicated zinc-sensitive mutants (Δ*cpxR*, Δ*pstB*, Δ*zntA*, Δ*zntR*) for 1 h, followed by gentamicin exclusion. Cells were lysed and intracellular bacterial loads were determined at 2 and 24 hpi. Data (mean + SEM, *n* = 4–5) are combined from four (*A*) or five (*B*) experiments. (*C*) HMDM were infected, as above, with wild-type MG1655 or corresponding *zntA* and *zntR* mutant strains. Data (mean + SEM, *n* = 5) are from five experiments (*P < 0.05, one-way ANOVA, with Dunnett’s multiple comparisons test). Assays for *B* and *C* were performed in parallel within the same experiments to enable direct comparisons between EC958 and MG1655. (*D*) HMDM were infected, as above, with wild-type MG1655, alongside Δ*cpxR*, Δ*pstB*, and Δ*zntA* MG1655 mutants. Cells were lysed and intracellular bacterial loads were determined at 4, 8, and 24 hpi. Data (mean + SEM, *n* = 3) are combined from three independent experiments.

Given evidence that EC958 was subjected to zinc stress within macrophages (Fig. 1*C*), we anticipated that EC958 mutants that displayed increased zinc sensitivity would be compromised for intramacrophage survival. We therefore began by profiling defined mutants of *cpxR* and *pstB* (top hits in our screen) (*SI Appendix*, Table S1) in intramacrophage survival assays. Surprisingly, we observed no difference in intracellular bacterial loads between wild-type EC958 and these mutants (Fig. 3*A*). Given this finding, we next directly compared defined mutants of *zntA* and *zntR* in both EC958 and the K-12 strain MG1655 in macrophage killing assays, because these genes have well-defined roles in defending against zinc toxicity. Whereas deletion of *zntA* or *zntR* in EC958 had no significant effect on bacterial survival within HMDM at 24 hpi (Fig. 3*B*), intramacrophage bacterial loads of the corresponding MG1655 mutants were significantly reduced (Fig. 3*C*). We further analyzed several MG1655 zinc-sensitive mutants (*pstB*, *cpxR*, *zntA*) over an infection time course, finding that all of these mutants were compromised for survival within macrophages compared with wild-type MG1655 (Fig. 3*D*). These findings confirm that macrophages effectively deploy zinc toxicity against a nonpathogenic *E. coli* strain, and suggest that EC958 is able to subvert this response.

### Generation and Validation of a Zinc Stress UPEC Reporter Strain.

The observation that zinc-sensitive EC958 mutants were not compromised for intramacrophage survival might be a consequence of resistance to zinc toxicity, evasion of zinc toxicity, or a combination of both mechanisms. We first considered the possibility that induced *zntA* mRNA in EC958 within macrophages (Fig. 1*C*) may reflect the response of only a subpopulation of bacteria, and that the majority of intracellular UPEC evades the zinc toxicity response. Subcellular localization of zinc can be monitored using fluorescent probes, such as FluoZin3-AM; however, these do not directly measure zinc stress (37). We therefore addressed this question by designing and validating EC958 strains that provide a more direct readout of zinc stress, and that enable tracking of this antimicrobial response at the single-cell level.

**Fig. 4.**
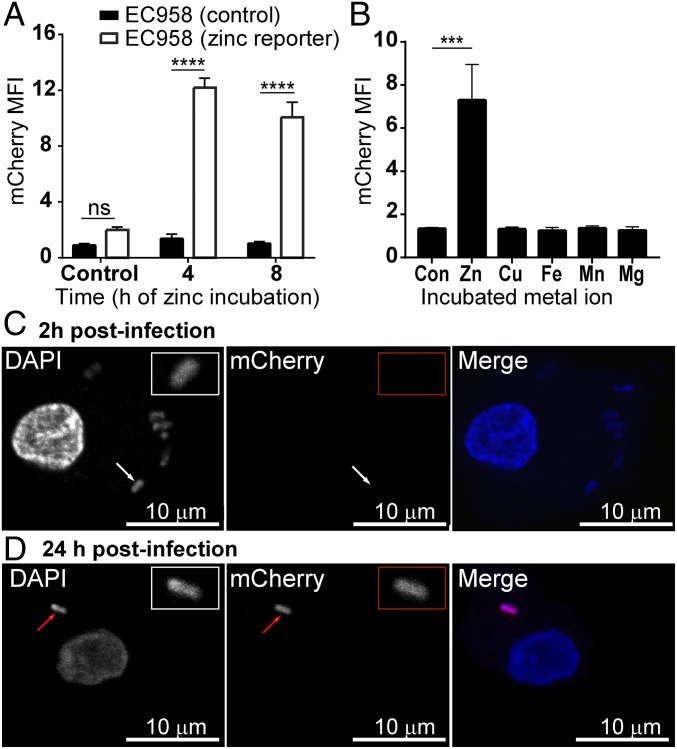
Development and validation of a UPEC EC958 zinc stress reporter strain. (*A*) Wild-type EC958 (filled bars) or the plasmid-encoded zinc stress reporter strain EC958 pQF50-zntA-mCherry (unfilled bars) were incubated in complete RPMI media (±500 µM ZnSO_4_) for 4 or 8 h, after which median mCherry fluorescence was determined by flow cytometry. Data (mean + SEM, *n* = 3) are combined from three experiments (****P < 0.0001, ns = not significant, two-way ANOVA with Sidak’s multiple comparisons test). (*B*) EC958-pQF50-zntA-mCherry was incubated in complete RPMI media supplemented with 500 µM of ZnSO_4_, CuSO_4_, FeSO_4_, MnCl_2_, or MgCl_2_ for 4 h, after which mCherry levels were assessed by flow cytometry. Data (mean + SEM, *n* = 3) are combined from three experiments (***P < 0.001, one-way ANOVA, with Dunnett’s multiple comparisons test). (*C* and *D*) HMDM were infected with EC958 pQF50-zntA-mCherry at an MOI of 100 for 1 h, followed by gentamicin exclusion. At 2 (*C*) or 24 (*D*) hpi, cells were fixed and stained with DAPI, and imaged for mCherry and DAPI fluorescence (white arrows: mCherry^−^ bacteria; red arrows: mCherry^+^ bacteria). All images were captured using a Zeiss LSM 710 upright confocal microscope at 60× magnification. Images are from a single experiment, and are representative of three independent experiments.

We first linked the *zntA* promoter to fluorescent reporters, using both chromosomal insertion and plasmid-based approaches (*SI Appendix*, Fig. S4). We then profiled the time course and specificity of inducible GFP expression for the chromosomal insertion strain (*SI Appendix*, Fig. S5 *A* and *B*); however, we ultimately found that the plasmid-based approach proved to be far more sensitive and robust (*SI Appendix*, Fig. S5 *C* and *D*). Although these initial studies investigated GFP reporters, we subsequently observed that corresponding mCherry reporter strains were more sensitive for macrophage infection assays. Zinc induced mCherry expression in the plasmid reporter strain EC958 pQF50-zntA-mCherry (Fig. 4*A*), and this response was again highly selective for zinc (Fig. 4*B*). This optimized strain (EC958 pQF50-zntA-mCherry) was thus used to assess the zinc stress response within macrophages. Consistent with the late-stage response of inducible *zntA* mRNA expression within HMDM (Fig. 1*C*), we detected mCherry within these cells at 24 hpi but not 2 hpi (Fig. 4 *C* and *D*). These data visually confirm that intracellular EC958 are subjected to a late-stage zinc stress response in human macrophages.

### EC958 Evades the Zinc Toxicity Response of Macrophages.

**Fig. 5.**
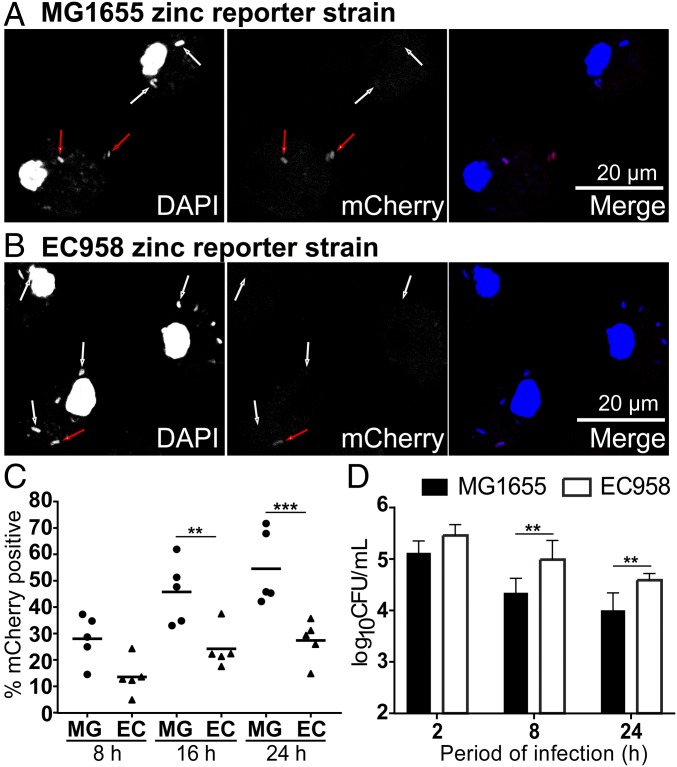
Analysis of zinc reporter strains demonstrates that EC958 evades the macrophage-mediated zinc stress response. (*A*–*C*) HMDM were infected with MG1655 pQF50-zntA-mCherry or EC958 pQF50-zntA-mCherry strains (MOI of 100) for 1 h, followed by gentamicin exclusion. At 8, 16, or 24 hpi, cells were fixed and stained with DAPI, then imaged for mCherry and DAPI fluorescence. Representative images at 16 hpi for MG1655 pQF50-zntA-mCherry and EC958 pQF50-zntA-mCherry are displayed in *A* and *B*, respectively (white arrows: mCherry^−^ bacteria; red arrows: mCherry^+^ bacteria). Data are from a single experiment and are representative of five experiments. (*C*) The percentage of mCherry^+^ bacteria (as determined by DAPI fluorescence) was recorded for multiple images taken at each time point, and an average value for MG1655 (MG) and EC958 (EC) was determined at 8, 16, and 24 hpi. Data (*n* = 5) are combined from five experiments (performed in parallel for MG1655 and EC958). (*D*) HMDM were infected with MG1655 or EC958 for 1 h, then extracellular bacteria were removed by gentamicin exclusion. Cells were lysed at 2, 8, and 24 hpi and the CFU/mL determined. Data (mean + SEM, *n* = 5) are combined from five experiments. Data were log_10_-transformed and analyzed utilizing a two-way ANOVA, with Sidak’s multiple comparisons test. For *C* and *D*, **P < 0.01 and ***P < 0.001.

To investigate mechanisms contributing to differential susceptibility of zinc-sensitive EC958 versus MG1655 mutants to killing by macrophages (Fig. 3), we next generated a corresponding MG1655 zinc stress reporter strain (MG1655 pQF50-zntA-mCherry). We confirmed that the EC958 and MG1655 reporter strains were similarly sensitive in their response to zinc (*SI Appendix*, Fig. S6*A*). We also found that the MG1655 reporter did not respond to copper over a concentration range (*SI Appendix*, Fig. S6*B*). The two reporter strains thus display similar sensitivity and specificity in reporting zinc stress, and we next used them for direct comparisons of the intramacrophage zinc stress response. By comparison with MG1655 pQF50-zntA-mCherry, there were many more intracellular EC958 pQF50-zntA-mCherry within HMDM at late time points that remained mCherry^−^ (Fig. 5 *A* and *B*). A blinded analysis revealed that substantially fewer intracellular EC958 were subjected to zinc stress at all of the time points examined (Fig. 5*C*). Consistent with this finding, intracellular bacterial loads of EC958 in HMDM were ∼six- and fourfold greater than were observed for MG1655 at 8 and 24 hpi, respectively (Fig. 5*D*). Collectively, these data suggest that evasion of macrophage-mediated zinc toxicity by EC958 enables increased intracellular survival within macrophages.

**Fig. 6.**
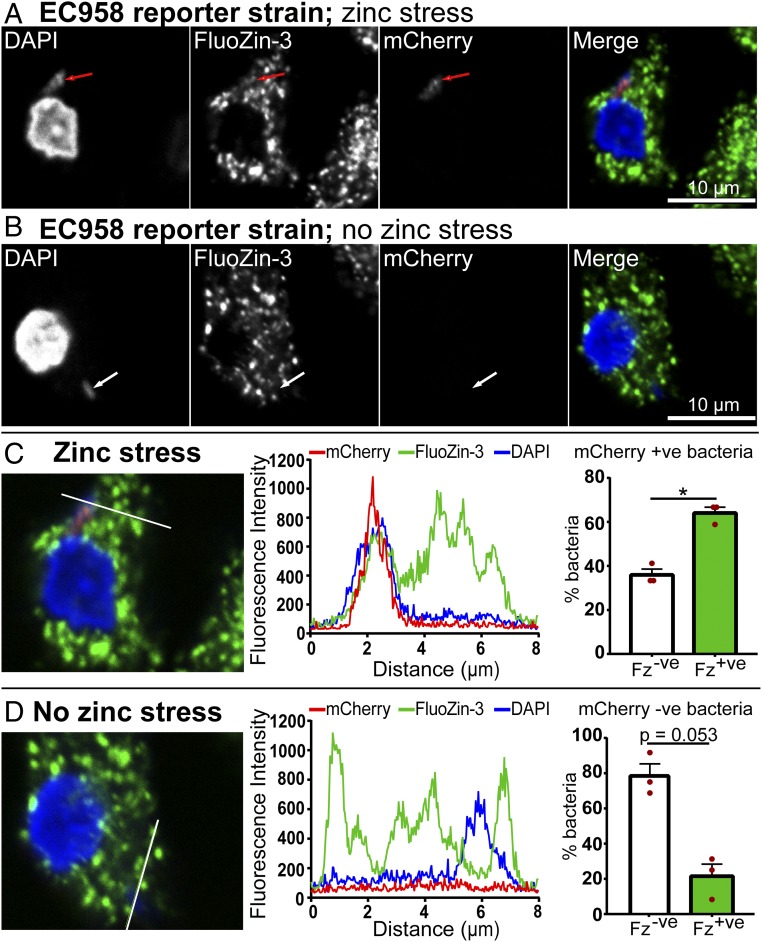
Zinc vesicles colocalize with EC958 that are experiencing zinc stress. (*A*–*D*) HMDM were infected with the EC958 pQF50-zntA-mCherry strain at an MOI of 100 for 1 h, followed by gentamicin exclusion. At 8, 16, or 24 hpi, cells were fixed and stained with DAPI and FluoZin-3, then imaged for mCherry, DNA, and zinc (FluoZin-3). Representative images at 24 hpi for mCherry^+^ and mCherry^−^ EC958 pQF50-zntA-mCherry are displayed in *A* and *B*, respectively (white arrows: mCherry^−^ bacteria; red arrows: mCherry^+^ bacteria). Data are from a single experiment and are representative of three experiments. (*C* and *D*) For single-plane images from *A* and *B*, respectively (magnification: 63×), fluorescence intensity of mCherry, FluoZin-3, and DAPI were assessed over a linear segment (histograms, *Center*), as indicated by white lines (*Left*). Images are from one experiment and are representative of three independent experiments. Images were also analyzed using a custom written, user-interactive ImageJ macro, enabling mCherry^+^ and mCherry^−^ bacteria to be assigned to “no colocalization” (unfilled bars = Fz^−^, graphical data, *Right*) or “colocalization” (green-filled bars = Fz^+^, graphical data, *Right*) with a FluoZin-3-marked zinc vesicle. The percentage colocalization was then determined for each experiment (with at least four images quantified in each experiment). Data (mean + SEM, *n* = 3) are combined from three experiments, and were analyzed using a paired *t* test (*P < 0.05).

To further investigate mechanisms by which EC958 evades the zinc stress response, we next used inductively coupled optical emission spectrometry (ICP-OES) to determine the total zinc content of control and *E. coli*-infected macrophages at 8 hpi. Using the approximate volume of a tissue-resident macrophage, 4,990 µm^3^, calculated by Krombach et al. (38), the concentration of zinc within resting macrophages was estimated to be ∼1.78 µg/mL (27.2 µM), with this modestly increasing to ∼2.28 µg/mL (34.80 µM) or ∼2.30 µg/mL (35.20 µM) in macrophages infected with MG1655 or EC958, respectively (*SI Appendix*, Fig. S7). This increase suggests that zinc uptake may contribute to the zinc toxicity response of macrophages. However, given that there was no obvious difference in the zinc content of macrophages responding to each of these *E. coli* strains, it seems unlikely that EC958 evades the zinc toxicity response by preventing zinc uptake or by promoting its export by macrophages. In accordance with this, when we infected HMDM with EC958 pQF50-zntA-mCherry and performed FluoZin3-AM staining for intracellular zinc (17), we still observed zinc-containing vesicles (Fig. 6 *A* and *B*). Moreover, we also observed that the small percentage of EC958 that were mCherry^+^ primarily colocalized with FluoZin3-AM staining (Fig. 6*C*, *Left* and *Center*), whereas mCherry^−^ EC958 did not (Fig. 6*D*, *Left* and *Center*). Unbiased image quantification across multiple independent experiments confirmed this differential staining pattern (Fig. 6 *C* and *D*, *Right*). These data are thus consistent with the existence of a substantial intracellular pool of EC958 that evades the macrophage zinc toxicity response.

**Fig. 7.**
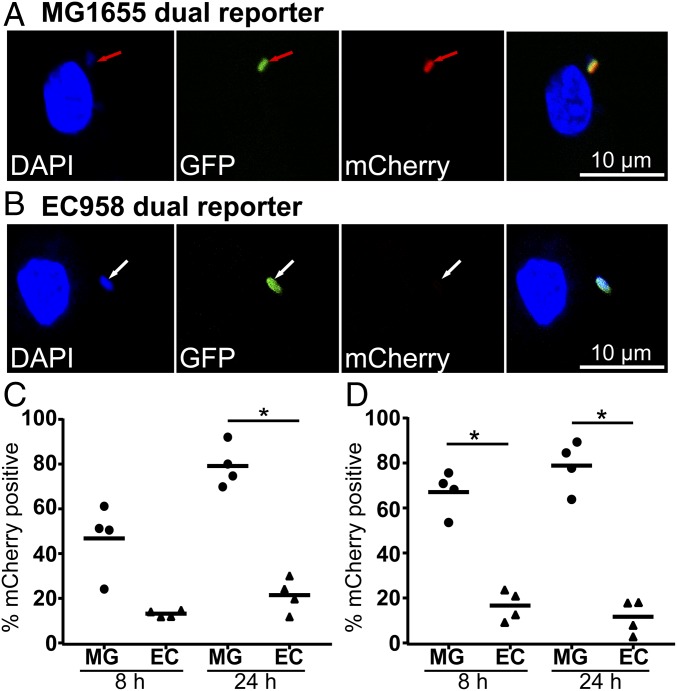
A dual reporter system confirms that EC958 evades the zinc stress response of human and mouse macrophages. (*A*–*C*) HMDM or (*D*) BMM were infected with MG1655 pGcCzntAp (MG) or EC958 pGcCzntAp (EC) (MOI of 100) for 1 h, followed by gentamicin exclusion. At 8 or 24 hpi, cells were fixed and stained with DAPI, then imaged for mCherry, DAPI, and GFP. The percentage of mCherry^+^/GFP^+^ viable bacteria was recorded for multiple images taken at each time point, and an average value for MG1655 and EC958 was determined at 8 and 24 hpi *A* and *B* are representative images of HMDM infected with (*A*) MG1655 or (*B*) EC958 at 8 hpi (red arrows: mCherry^+^ bacteria; white arrows: mCherry^−^ bacteria). Data in *C* and *D* are combined from four independent experiments (horizontal bars indicate mean, *P < 0.05, one-way ANOVA with Sidak’s multiple comparisons test). Assays were performed in parallel for MG1655 and EC958.

It was conceivable that the reduced percentage of EC958 subjected to zinc stress within HMDM compared with MG1655, as determined by *zntA*-dependent mCherry expression (Fig. 5*C*), could simply reflect increased EC958 killing and loss of mCherry. However, on the basis of intracellular survival data (Fig. 5*D*), this seemed unlikely. To exclude this possibility, we next constructed a dual reporter system that possesses on a single plasmid (pGcCzntAp), constitutively expressed GFP and inducibly expressed mCherry under the control of the *zntA* promoter. Using this tool, we observed that most MG1655 within HMDM were both GFP^+^ and mCherry^+^, whereas the majority of EC958 expressed GFP but not mCherry (Fig. 7 *A* and *B*). Blinded image quantification revealed that, for GFP^+^ bacteria within HMDM at 24 hpi, ∼80% of MG1655 and ∼25% of EC958 were mCherry^+^ (Fig. 7*C*). Moreover, most mCherry^−^ MG1655 detected by DAPI within HMDM were also GFP^−^. We conclude that these represent nonviable bacteria, whereas the GFP^+^/mCherry^−^ intracellular EC958 pool likely resides within a niche not subjected to zinc toxicity. This result in HMDM was mirrored in murine bone marrow-derived macrophages (BMM), with ∼80% of GFP^+^ MG1655 being mCherry^+^ at 24 h, compared with ∼20% of EC958 (Fig. 7*D*).

### EC958 Possesses Enhanced Zinc Tolerance Compared with MG1655.

While the above data support a model in which EC958 evades the macrophage zinc toxicity response, ∼20% of EC958 cells were still subjected to zinc stress within macrophages (Fig. 7 *C* and *D*). We therefore considered whether EC958 also has a capacity to tolerate higher zinc concentrations than MG1655, because the latter is more effectively cleared by human macrophages (Fig. 5*D*). Indeed, the growth of MG1655 in 1.25 mM zinc was substantially impaired compared with EC958 (*SI Appendix*, Fig. S8*A*). This finding suggests that zinc tolerance could contribute to intracellular survival of the small pool of EC958 that are unable to escape the macrophage zinc toxicity response (Fig. 7 *C* and *D*). We also observed a difference in zinc tolerance between the EC958 and MG1655 *zntA* mutants during growth in the presence of 0.6 mM zinc (*SI Appendix*, Fig. S8*B*), suggesting that this difference is at least in part due to intrinsic factors that enable EC958 to tolerate higher levels of zinc stress. In total, these data indicate that EC958 is equipped with the capacity to tolerate innate immune-mediated zinc stress, in addition to its ability to deploy evasion of the macrophage zinc toxicity response.

### EC958 Both Evades and Resists Immune-Mediated Zinc Toxicity in Vivo.

**Fig. 8.**
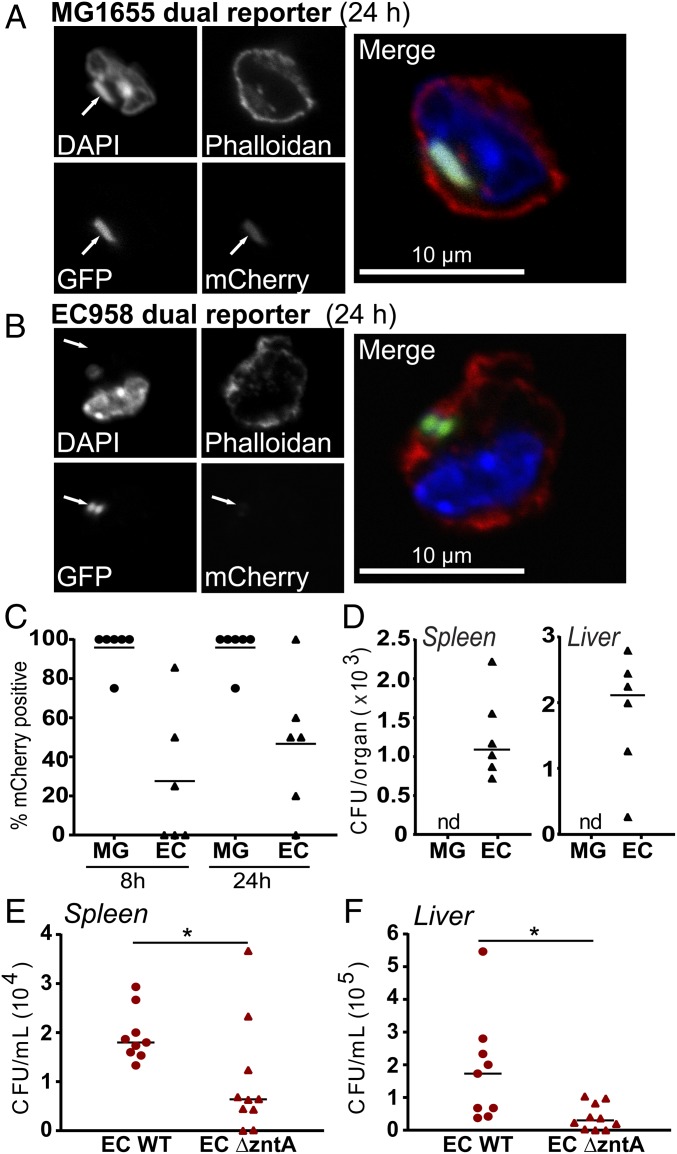
EC958 both evades and resists zinc toxicity after intraperitoneal challenge, enabling dissemination to the liver and spleen. Mice were intraperitoneally infected with 10^6^ CFU of either the MG1655 pGcCzntAp (MG) or EC958 pGcCzntAp (EC) dual reporter strains, after which peritoneal exudates, spleens and livers were collected at 8 and 24 hpi. Representative images from infiltrating peritoneal cells at 24 hpi containing (*A*) MG1655 or (*B*) EC958, stained with DAPI (nucleus) and Phalloidan-647 (actin). (Magnification: 63×.) (*C*) For each bacterial strain, the percentage of GFP^+^ bacteria that were also mCherry^+^ was calculated in peritoneal exudates (horizontal bars indicate mean, *n* = 6 mice per group from two independent experiments). (*D*) Spleens and livers from infected mice were collected and chloramphenicol-resistant CFU/organ were determined (horizontal bar indicated median, *n* = 6 mice per group). nd, not detectable. (*E* and *F*) Mice were intraperitoneally infected with 10^7^ CFU of either the wild-type EC958 or EC958 *ΔzntA*, after which spleens and livers were collected at 20 hpi. Organs were homogenized and the CFU/organ were determined (horizontal bars indicate median, *n* = 9 mice from two independent experiments). *P < 0.05, Welch’s *t* test.

Given that EC958 evades zinc toxicity within murine macrophages (Fig. 7*D*), we next examined whether this phenomenon was also apparent in a mouse model. ST131 and other clones belonging to the B2 *E. coli* phylogenetic group are overrepresented in patients with spontaneous bacterial peritonitis (39). We therefore used our dual reporter system in an intraperitoneal challenge model to monitor zinc stress to MG1655 versus EC958 at 8 and 24 h. Whereas almost all MG1655 were subjected to zinc stress (GFP^+^/mCherry^+^) (Fig. 8*A*), many of the intracellular EC958 were not (GFP^+^/mCherry^−^) (Fig. 8*B*). Quantification of the zinc stress response for MG1655 and EC958 in peritoneal exudates of infected mice is shown in Fig. 8*C*. We also found that EC958 colonized both the spleen and liver after intraperitoneal challenge, whereas MG1655 was unable to disseminate (Fig. 8*D*). This disparity between the two strains in systemic spread is consistent with the striking difference in evasion of the innate immune zinc toxicity response by EC958 versus MG1655, although other mechanisms are also likely to contribute. Interestingly, in contrast to the consistency observed in evasion of intramacrophage zinc toxicity in vitro (∼80% EC958 in both HMDM and BMM were mCherry^−^) (Fig. 7 *C* and *D*), we observed much greater variability between individual mice in the intraperitoneal challenge model (Fig. 8*C*). Given this, we considered that UPEC may also use resistance to defend against innate immune-mediated zinc toxicity in vivo. We therefore assessed dissemination of wild-type EC958 and our EC958 *zntA* mutant, finding that bacterial loads for the latter were significantly reduced in both the liver and spleen (Fig. 8 *E* and *F*). Collectively, the above data suggest that the capacity of EC958 to both evade and resist innate immune-mediated zinc toxicity contributes to host colonization and systemic spread.

## Discussion

We, and others, have shown that human macrophages deploy zinc toxicity as an antimicrobial strategy against the intramacrophage bacterial pathogens *S*. Typhimurium (17) and *M*. *tuberculosis* (16). Recently, the zinc toxicity antibacterial pathway was shown to be operational in the soil amoeba *Dictyostelium discoideum* (40), thus indicating that this is an evolutionarily conserved innate immune response. Here we show, through the use of newly developed zinc stress reporter systems as well as zinc-sensitive mutants, that UPEC strain EC958 both evades and resists innate immune-mediated zinc toxicity in vitro and in vivo. Despite EC958 up-regulating transcription of *zntA* during human macrophage infection (Fig. 1*C*), zinc-sensitive EC958 mutants (*cpxR*, *pstB*, *zntA*, *zntR*) were not more susceptible to clearance by macrophages, compared with the wild-type strain (Fig. 3 *A* and *B*). The reduced intramacrophage survival of corresponding mutants in MG1655 under the same experimental systems (Fig. 3 *C* and *D*) implied that EC958 employs strategies to resist or avoid zinc toxicity.

Microscopic analysis revealed that only a small proportion (∼20%) of intracellular EC958 experience zinc stress during human macrophage infection, as opposed to the majority (∼80%) of intracellular MG1655 (Fig. 7 *C* and *D*). The EC958 and MG1655 zinc reporter strains were not responsive to copper or other biologically relevant metal ions (Fig. 4*B* and *SI Appendix*, Fig. S6*B*), and showed similar zinc sensitivity (*SI Appendix*, Fig. S6*A*). In fact, if anything, the EC958 reporter strain was slightly more sensitive, so one should expect slightly higher reporter activity for this strain within macrophages. We thus conclude that the difference between the two *E. coli* strains in zinc reporter activity within macrophages reflects evasion of innate immune-mediated zinc toxicity by EC958. This conclusion is supported by our observation that ∼80% of those intramacrophage EC958 that did not report zinc stress did not colocalize with zinc, as assessed by FluoZin-3 staining (Fig. 6*D*). Differential zinc reporter activity for MG1655 versus EC958 was also observed within infiltrating cells of the mouse peritoneal cavity following infection (Fig. 8 *A* and *B*), although in this case, the response was more heterogeneous for EC958 (Fig. 8*C*). In addition to demonstrating that this host subversion strategy is conserved in humans and mice, these findings highlight the utility of these reporter strains for tracking zinc toxicity and evasion of this response in vivo. Given that *S*. Typhimurium also evades the macrophage zinc toxicity response (17), subversion of this pathway may be a common feature of pathogenic Gram-negative bacteria that contributes to their survival within these long-lived innate immune cells.

Previous studies have examined bacterial mutants defective in zinc export systems to demonstrate a role for zinc toxicity as an innate immune antimicrobial response. During infection of human macrophages, for example, *M. tuberculosis* up-regulates genes encoding heavy metal P-Type ATPases, including the CtpC zinc efflux pump (16). Accordingly, a *ctpC* mutant exhibited reduced intramacrophage survival, consistent with the accumulation of zinc within bacteria-containing phagosomes during *M. tuberculosis* infection. Similarly, deletion of *czcD*, which encodes a zinc exporter, rendered *Streptococcus pyogenes* highly susceptible to killing by neutrophils (18), with zinc mobilized toward engulfed bacteria from stores in lysosomes and azurophilic granules (19). In both instances, effective zinc efflux was required for resistance of these pathogens to innate immune-mediated killing. Our studies with the *E. coli* K-12 strain MG1655 add to this literature, revealing that zinc efflux systems are also important for intramacrophage survival of nonpathogenic *E. coli* (Fig. 3 *C* and *D*). The use of zinc toxicity by innate immune cells would therefore appear to be a general response to bacterial infection, consistent with our previous observation that multiple TLR ligands initiate zinc-containing vesicle formation within macrophages (17).

*S*. Typhimurium evades macrophage-mediated zinc toxicity via SPI-1, but deletion of *zntA* on a ∆SPI-1 background did not further reduce intracellular survival of *S*. Typhimurium, in comparison with the ∆SPI-1 mutant alone (17). Although zinc efflux systems were subsequently shown to be important for host colonization by this intramacrophage pathogen in vivo (41), the above finding suggests that *S*. Typhimurium employs additional mechanisms to counter macrophage-mediated zinc toxicity. The same is also likely to apply to UPEC, because ∼20–30% of intracellular EC958 still experienced zinc stress within HMDM (Fig. 7 *C* and *D*). Consistent with this hypothesis, we also found that EC958 was more tolerant than MG1655 to zinc (*SI Appendix*, Fig. S8*A*). The fact that a similar phenomenon was also observed in the EC958 versus MG1655 *zntA* mutants (*SI Appendix*, Fig. S8*B*) suggests that this discrepancy is due to intrinsic differences in the capacity of these two strains to tolerate zinc stress. This interpretation is consistent with our TraDIS analysis, because all of the genes identified in our screen are conserved between EC958 and MG1655. This increased tolerance to zinc is likely important for combating innate immune-mediated zinc toxicity in vivo, given the reduced colonization of the spleen and liver by an EC958 *zntA* mutant (Fig. 8 *E* and *F*). The capacity of EC958 to evade the zinc toxicity response in vivo (Fig. 8*C*) was more variable than was observed in vitro (Fig. 7 *C* and *D*). It is possible that inflammatory mediators present in vivo can overcome EC958-mediated evasion of macrophage-mediated zinc toxicity and that other cell types, such as neutrophils, more effectively utilize this antimicrobial response against UPEC. Given that neutrophils produce high levels of reactive oxygen species, the observation that paraquat increased the sensitivity of an EC958 *zntA* mutant to zinc (*SI Appendix*, Fig. S3) supports the view that zinc resistance may be important for combating antimicrobial responses of these cells.

With an increasing appreciation of the role of zinc toxicity in innate immune antimicrobial responses (13, 16–18), an understanding of the mechanisms by which bacterial pathogens tolerate zinc stress is clearly required. Several recent studies have shed light on mechanisms by which zinc exerts cytotoxic effects on both Gram-positive (20, 42) and Gram-negative (22, 43) bacterial pathogens. Our functional genetic approach involved the application of forefront TraDIS technology (Fig. 2), and allowed us to define the full suite of EC958 genes involved in zinc resistance. While several of these genes are known to confer zinc-resistance (e.g., *zntA*, *zntR*) (24, 33) or have been linked to stress responses (e.g., *cpxR*, *ompR*) (44, 45) in *E. coli*, others are yet to be explored in the context of defense against zinc stress. For example, our data indicate that mutants that have reduced cell envelope integrity (e.g., *ldcA*, *yciB*, *tolB*) and defective cell division (e.g., *minC*, *minD*, *ftsX*) are particularly susceptible to zinc stress. Previous work has shown that exposure to some metal ions can induce modifications in LPS via the PmrA/PmrB two-component regulatory system and this leads to increased resistance against antimicrobial peptides (46). In *E. coli* K-12, exposure to zinc induces transcription of *pmrAB* (previously referred to as *basRS*) (47) and thus it remains to be determined if some of the genes associated with membrane integrity that were identified in our screen are linked to the PmrA/PmrB regulatory pathway.

The TraDIS screen also confirmed the established link between zinc and phosphate transport. The *pitA* mutant displayed enhanced resistance to zinc, consistent with a previous report in *E. coli* K-12 (34). PitA is a low-affinity phosphate transporter in contrast to the Pst transporter, which is a high-affinity transporter inducible in low phosphate (48). The *pstB* gene was the most significant hit in our TraDIS screen (*SI Appendix*, Table S1), and its mutation in EC958 led to increased zinc sensitivity (Fig. 2*C*). This may be explained by the fact that, in a *pstB* mutant, phosphate uptake would depend upon the Pit system, resulting in increased sensitivity to zinc. We predicted that zinc may act synergistically with other innate immune antimicrobial factors and thus tested the combinatorial effect of zinc and cathelicidin or reactive oxygen species on *E. coli* survival. While our experiments did not reveal a synergistic effect between zinc and cathelicidin, we did observe that the superoxide anion generator paraquat sensitized an EC958 *zntA* mutant to zinc (*SI Appendix*, Fig. S3). Given this result, and the recent finding that host-derived free radicals cause zinc stress in *S*. Typhimurium (49), it would be of interest in future studies to examine whether any of the zinc-sensitive mutants identified in our TraDIS screen are more sensitive to neutrophils, as well as other innate immune mechanisms of zinc toxicity.

It is now clear that the capacity of UPEC to occupy intracellular niches is an important phenotype in its pathogenic lifestyle. For example, intracellular bacterial community formation within bladder epithelial cells in experimental mice contributes to innate immune evasion during the early stages of acute UTI (4, 5). Within this environment, UPEC evades neutrophil killing and suppresses the release of host-protective inflammatory mediators (50–53). Whether bladder epithelial cells also engage an inducible zinc toxicity response that is subverted by UPEC is unknown at this stage. In addition to replicating within epithelial cells, some UPEC strains can also persist within primary mouse and human macrophages (12). Here we report that EC958 also has a propensity to survive within macrophages, in comparison with MG1655 (Fig. 5*D*). The capacity to survive within these cells is predicted to provide an intracellular niche for UPEC to enable colonization and dissemination, for example during accession to the kidneys and translocation into the bloodstream. This conclusion is supported by our findings from an intraperitoneal challenge model, in which EC958, but not MG1655, disseminated to the spleen and liver of infected mice (Fig. 8*D*). However, we note that other factors, beyond intramacrophage survival and evasion of zinc toxicity by EC958, are likely to contribute to the difference between these strains in their capacity to spread systemically. A previous study showed that CFT073 disseminates to multiple organs after intravenous administration (54), but to our knowledge, this demonstration of systemic spread of a UPEC strain after intraperitoneal challenge is unique. The clinical relevance of our findings is reflected by the observation that ST131 and other B2 phylogeny group *E. coli* strains are commonly found in ascites fluid of patients with spontaneous bacterial peritonitis (39), and such patients are at risk for systemic spread and severe sepsis (55–57). Indeed, an analysis of hospital-admitted patients with Gram-negative bacterial infections in the bloodstream found that ST131 was highly overrepresented (58). We thus propose that intraperitoneal challenge of C57BL/6 mice with UPEC will provide a useful model to understand mechanisms involved in systemic spread and sepsis in patients with spontaneous bacterial peritonitis.

The roles of transition metals, in particular copper and zinc, have been examined in the context of UPEC infection (59, 60). UPEC copper-responsive genes were up-regulated during experimental UTI, and mutation of the *cus* genes in the UPEC reference strain CFT073 resulted in significantly decreased colonization of the bladder and kidneys (61). Copper also appears to play an important role in human UTI; UPEC copper-responsive genes are up-regulated during disease (61), and infection by UPEC triggers elevation of copper levels (61, 62). We note, however, that the zinc reporter strains developed here are not responsive to copper (Fig. 4*B* and *SI Appendix*, Fig. S6*B*). Although we demonstrate that UPEC are exposed to zinc toxicity in the intramacrophage environment, others have shown that the extracellular environment of the bladder lumen may be limited for zinc. The ZnuABC zinc uptake system was essential for UPEC colonization of the bladder and kidney in experimental mice (63). Furthermore, transcriptome analysis of UPEC in samples collected directly from UTI patients indicates that genes associated with zinc uptake (*znuABC*, *zinT*, *zupT*) are highly expressed during human infection (62, 64). Taken together, current evidence would therefore suggest that UPEC are likely to be subjected to both zinc toxicity and zinc starvation, depending on the specific microenvironment. Recent research provides evidence for such a mechanism during infection with *Streptococcus*, which experiences both extracellular zinc starvation as a consequence of calprotectin-mediated zinc sequestration, as well as intracellular zinc toxicity mediated by neutrophils (19). An understanding of the specific UPEC factors that enable evasion of innate immune-mediated zinc stress is now required to determine the contribution of this subversion mechanism to survival within macrophages and epithelial cells, as well as virulence. Because the zinc content of EC958- versus MG1655-infected macrophages was similar (*SI Appendix*, Fig. S7), it seems unlikely that the evasion mechanism involves inhibition or promotion of zinc uptake or export, respectively. Rather, the fact that most intramacrophage EC958 that had not experienced zinc stress also did not colocalize with zinc (Fig. 6*D*) supports the view that this pathogen is able to subvert trafficking pathways that deliver antimicrobial zinc.

In this study, we describe a range of plasmid-based zinc stress reporter systems, and validated that these specifically report exposure to high levels of zinc. We note that the concentrations of zinc to which these strains respond (>50 µM) (*SI Appendix*, Figs. S5*D* and S6*A*) are relevant to the concentrations likely to be experienced during innate immune attack. For example, we estimate using ICP-OES that the average zinc concentration within *E. coli*-infected macrophages at the time in which the zinc toxicity response is deployed is ∼35 µM (*SI Appendix*, Fig. S7). Within the concentrated environment of zinc-containing vesicles, one would anticipate a much higher zinc concentration than this. Indeed, others have reported that the zinc concentration within the phagosome of *M. tuberculosis*-infected macrophages can reach ∼500 µM by 24 hpi, and ∼1,800 µM in *Mycobacterium avium*-infected macrophages treated with tumor necrosis factor (65). We therefore conclude that the concentrations of zinc used to validate zinc stress reporter strains, and to identify zinc-resistance genes in UPEC, are relevant to macrophage-mediated zinc toxicity.

Unlike other analyses that are performed at a population level, for example quantitative real-time PCR (Fig. 1*C*), these zinc reporter systems make it possible to pinpoint, in a single bacterial cell within an immune cell, whether zinc toxicity has been deployed. A key advantage of this approach is that it relies on a readout of zinc stress from the bacterial cell, as opposed to a shift in the concentration of zinc, which may or may not correlate with an antimicrobial effect. For example, the presence of other nutrients or metal ions in a specific microenvironment would impact whether altered zinc concentrations will exert detrimental (or beneficial) effects on an infectious agent. Thus, while measurements of changes in zinc concentrations in both in vitro and in vivo infection models have been highly informative (66–68), we predict that the use of these zinc stress reporter systems will, in the future, provide further insights into innate immune-mediated zinc toxicity at the molecular, cellular, and organismal level. In summary, this study has defined the zinc resistome in EC958, and has shown that this clinically relevant UPEC strain both evades and resists innate immune-mediated zinc toxicity. More broadly, it expands our understanding of regulated zinc trafficking as an antimicrobial weapon of macrophages, and has validated the use of zinc stress reporter systems both in vitro and in vivo as important new tools that can now be applied to understand molecular processes involved in both host engagement and pathogen evasion of the innate immune zinc toxicity response.

## Experimental Procedures

Key methods used in this manuscript are provided below. Extended methods for generation of zinc reporter plasmids and strains, TraDIS, bacterial growth curves, quantitative real-time PCR, zinc and cathelicidin (LL-37) checkerboard assays, flow cytometry, and determination of zinc concentrations by ICP-OES are provided in the *SI Appendix*. All data generated in this study are available in the main text or the *SI Appendix*.

### Ethics Statement.

All work involving primary human cells was approved by the University of Queensland Medical Research Ethics Committee (certificate no. 2013001519). Work involving mice was approved by the University of Queensland Animal Ethics Committee (AEC approval nos. IMB/468/17 and IMB/118/15).

### Bacterial Strains and Culture Conditions.

Bacterial strains and reporter plasmids (*SI Appendix*, Fig. S9 and Table S3), oligonucleotides used to generate reporter plasmids, reporter strains, and mutants (*SI Appendix*, Table S4), and primers used to quantify bacterial gene expression (*SI Appendix*, Table S5) are listed in *SI Appendix*. All *E. coli* strains were routinely cultured at 37 °C on solid or in liquid LB medium supplemented with appropriate antibiotics [chloramphenicol (30 mg/L) or ampicillin (100 mg/L)]. Mutants in MG1655 and EC958 were made using the λ-red recombinase system, as previously described (31).

### Mammalian Cell Culture.

To generate HMDM, CD14^+^ monocytes were isolated from human buffy coats, obtained from the Australian Red Cross Blood Service. Peripheral blood mononuclear cells were isolated using Ficoll (GE Healthcare) density gradient separation followed by positive selection of monocytes (CD14^+^ magnetic beads), as previously described (17). Monocytes were then cultured in Iscove’s modified Dulbecco’s medium (Life Technologies) for 7 d to generate HMDM. HMDM media was supplemented with 10% heat-inactivated FBS, 50 U/mL penicillin, and 50 µg/mL streptomycin (Life Technologies), as well as 1 × 10^4^ U/mL recombinant human colony stimulating factor-1 (a gift from Chiron). Penicillin and streptomycin were excluded from cultures during bacterial infection assays. To generate mouse BMM, bone marrow cells from the femurs and tibias of C57BL/6 mice were cultured for 7 d in the presence of 1 × 10^4^ U/mL recombinant human colony stimulating factor-1, as previously described (69).

### Infection Assays.

Bacterial infection assays were performed as previously described (12). In brief, bacteria were grown in LB medium at 37 °C overnight and then used to infect cultured macrophages at a multiplicity of infection (MOI) of 100. After 1 h, the media was removed and replaced with media containing 200 µg/mL gentamicin for 1 h, before further culture in media containing 20 µg/mL gentamicin (gentamicin exclusion). To determine intracellular bacterial loads, cells were lysed with 0.1% Triton X-100 in PBS at 2, 4, 8, or 24 hpi and plated in triplicate onto LB agar.

### Confocal Microscopy.

Immunofluoresence imaging was performed using an Olympus BX-51, a Zeiss LSM 510 Meta inverted microscope and a Zeiss 710 LSM Meta fitted to an Axiovert 200 upright microscope running Zen Black 2012. Cells grown on coverslips in 24-well plates were washed twice with PBS and then fixed in 4% PFA at room temperature for 10 min. Nuclear DNA was stained with DAPI (Life Technologies) at a concentration of 1 μg/mL, and F-actin was stained with Phalloidan-647 (Cell Signaling) at a concentration of 16.5 nM. Intracellular zinc was detected by incubating fixed cells for 30 min with the zinc-binding fluorophore FluoZin-3 AM (Life Technologies) at a concentration of 5 µM, as previously described (17). Excitation of DAPI, GFP, and mCherry was achieved through laser emission of 405 nm, 488 nm, and 561 nm, respectively. For determination of bacteria/FluoZin-3 colocalization, confocal images were analyzed using a custom written, user-interactive ImageJ macro. Briefly, images were split into their respective channels, with the DAPI channel (Ch3) subjected to background subtraction and median filtering before being thresholded by the user. Segmented objects of 0.3- to 2-µm size were added to the region of interest manager and were used to measure the maximum and median fluorescent intensities of the remaining red and green channels (Ch1/Ch2). Measured data (CSV file output) was further analyzed using GraphPad Prism software. This script is available for public use via GitHub link https://github.com/NickCondon/Finding-Bacteria-and-measuring-all-three-channels.

### Intraperitoneal Infections.

C57BL/6 male mice at 8 wk of age were infected (intraperitoneally) with 10^6^ CFU of either MG1655 or EC958, or were administered 100 µL PBS intraperitoneally (base line control for microscopy). All mice were monitored according to ethics requirements, then killed at 8 or 24 h, after which peritoneal exudates, livers, and spleens were collected. Peritoneal exudates were centrifuged, counted, and plated at 300,000 cells per coverslip for 1 h, before being fixed and stained for 30 min with DAPI and Phalloidan-647. To determine bacterial loads in the spleen and liver, organs were homogenized in 1 mL PBS utilizing a T10 Ultra Turrax homogenizer (spleen: speed 3, 15 s; liver: speed 4, 1 min). Triton X-100 was added to a final concentration of 0.1%, with homogenates plated on LB plates supplemented with chloramphenicol (30 mg/L) and incubated overnight before counting of CFU to determine CFU per organ. In a second series of experiments, C57BL/6 male mice at 8 wk of age were infected by intraperitoneal challenge with 10^7^ CFU of either wild-type EC958 or the EC958 *zntA* mutant. Bacterial loads in spleen and liver were assessed at 20 hpi using the same method.

### Statistical Analyses.

Prism 5 software was used to perform statistical analyses on data compiled from at least three independent experiments. Specific statistical tests used are indicated in the individual figure legends.

## Supplementary Material

Supplementary File

## References

[1] Foxman B (2003). Epidemiology of urinary tract infections: Incidence, morbidity, and economic costs. Dis Mon.

[2] Harding GK, Ronald AR (1994). The management of urinary infections: What have we learned in the past decade?. Int J Antimicrob Agents.

[3] Petty NK (2014). Global dissemination of a multidrug resistant *Escherichia coli* clone. Proc Natl Acad Sci USA.

[4] Hannan TJ (2012). Host-pathogen checkpoints and population bottlenecks in persistent and intracellular uropathogenic *Escherichia coli* bladder infection. FEMS Microbiol Rev.

[5] Hunstad DA, Justice SS (2010). Intracellular lifestyles and immune evasion strategies of uropathogenic *Escherichia coli*. Annu Rev Microbiol.

[6] Abraham SN, Miao Y (2015). The nature of immune responses to urinary tract infections. Nat Rev Immunol.

[7] Schiwon M (2014). Crosstalk between sentinel and helper macrophages permits neutrophil migration into infected uroepithelium. Cell.

[8] Loughman JA, Hunstad DA (2011). Attenuation of human neutrophil migration and function by uropathogenic bacteria. Microbes Infect.

[9] Schaale K (2016). Strain- and host species-specific inflammasome activation, IL-1β release, and cell death in macrophages infected with uropathogenic *Escherichia coli*. Mucosal Immunol.

[10] M V Murthy A (2018). Regulation of hemolysin in uropathogenic *Escherichia coli* fine-tunes killing of human macrophages. Virulence.

[11] Carey AJ (2016). Uropathogenic *Escherichia coli* engages CD14-dependent signaling to enable bladder-macrophage-dependent control of acute urinary tract infection. J Infect Dis.

[12] Bokil NJ (2011). Intramacrophage survival of uropathogenic *Escherichia coli*: Differences between diverse clinical isolates and between mouse and human macrophages. Immunobiology.

[13] Stocks CJ, Schembri MA, Sweet MJ, Kapetanovic R (2018). For when bacterial infections persist: Toll-like receptor-inducible direct antimicrobial pathways in macrophages. J Leukoc Biol.

[14] Schaible UE, Kaufmann SHE (2004). Iron and microbial infection. Nat Rev Microbiol.

[15] Stafford SL (2013). Metal ions in macrophage antimicrobial pathways: Emerging roles for zinc and copper. Biosci Rep.

[16] Botella H (2011). Mycobacterial p(1)-type ATPases mediate resistance to zinc poisoning in human macrophages. Cell Host Microbe.

[17] Kapetanovic R (2016). *Salmonella* employs multiple mechanisms to subvert the TLR-inducible zinc-mediated antimicrobial response of human macrophages. FASEB J.

[18] Ong CL, Gillen CM, Barnett TC, Walker MJ, McEwan AG (2014). An antimicrobial role for zinc in innate immune defense against group A streptococcus. J Infect Dis.

[19] Ong CY, Berking O, Walker MJ, McEwan AG (2018). New insights into the role of zinc acquisition and zinc tolerance in group A streptococcal infection. Infect Immun.

[20] McDevitt CA (2011). A molecular mechanism for bacterial susceptibility to zinc. PLoS Pathog.

[21] Ong CL, Walker MJ, McEwan AG (2015). Zinc disrupts central carbon metabolism and capsule biosynthesis in *Streptococcus pyogenes*. Sci Rep.

[22] Xu FF, Imlay JA (2012). Silver(I), mercury(II), cadmium(II), and zinc(II) target exposed enzymic iron-sulfur clusters when they toxify *Escherichia coli*. Appl Environ Microbiol.

[23] Totsika M (2011). Insights into a multidrug resistant *Escherichia coli* pathogen of the globally disseminated ST131 lineage: Genome analysis and virulence mechanisms. PLoS One.

[24] Rensing C, Mitra B, Rosen BP (1997). The zntA gene of *Escherichia coli* encodes a Zn(II)-translocating P-type ATPase. Proc Natl Acad Sci USA.

[25] Wang D, Hosteen O, Fierke CA (2012). ZntR-mediated transcription of zntA responds to nanomolar intracellular free zinc. J Inorg Biochem.

[26] Achard ME (2012). Copper redistribution in murine macrophages in response to *Salmonella* infection. Biochem J.

[27] Achard ME (2010). The multi-copper-ion oxidase CueO of *Salmonella enterica* serovar Typhimurium is required for systemic virulence. Infect Immun.

[28] White C, Lee J, Kambe T, Fritsche K, Petris MJ (2009). A role for the ATP7A copper-transporting ATPase in macrophage bactericidal activity. J Biol Chem.

[29] Chaturvedi KS (2014). Cupric yersiniabactin is a virulence-associated superoxide dismutase mimic. ACS Chem Biol.

[30] Macomber L, Imlay JA (2009). The iron-sulfur clusters of dehydratases are primary intracellular targets of copper toxicity. Proc Natl Acad Sci USA.

[31] Phan MD (2013). The serum resistome of a globally disseminated multidrug resistant uropathogenic *Escherichia coli* clone. PLoS Genet.

[32] Hancock SJ (2017). Identification of IncA/C plasmid replication and maintenance genes and development of a plasmid multilocus sequence typing scheme. Antimicrob Agents Chemother.

[33] Brocklehurst KR (1999). ZntR is a Zn(II)-responsive MerR-like transcriptional regulator of zntA in *Escherichia coli*. Mol Microbiol.

[34] Beard SJ (2000). Evidence for the transport of zinc(II) ions via the pit inorganic phosphate transport system in *Escherichia coli*. FEMS Microbiol Lett.

[35] Li D (2014). Expression of the antimicrobial peptide cathelicidin in myeloid cells is required for lung tumor growth. Oncogene.

[36] Li D (2015). Cathelicidin, an antimicrobial peptide produced by macrophages, promotes colon cancer by activating the Wnt/β-catenin pathway. Oncotarget.

[37] Maret W (2015). Analyzing free zinc(II) ion concentrations in cell biology with fluorescent chelating molecules. Metallomics.

[38] Krombach F (1997). Cell size of alveolar macrophages: An interspecies comparison. Environ Health Perspect.

[39] Bert F (2010). Genetic diversity and virulence profiles of *Escherichia coli* isolates causing spontaneous bacterial peritonitis and bacteremia in patients with cirrhosis. J Clin Microbiol.

[40] Barisch C (2018). Localization of all four ZnT zinc transporters in *Dictyostelium* and impact of ZntA and B knockout on bacteria killing. J Cell Sci.

[41] Huang K (2018). Investigation of the role of genes encoding zinc exporters *zntA*, *zitB*, and *fieF* during *Salmonella* Typhimurium infection. Front Microbiol.

[42] Eijkelkamp BA (2014). Extracellular zinc competitively inhibits manganese uptake and compromises oxidative stress management in *Streptococcus pneumoniae*. PLoS One.

[43] Stähler FN (2006). The novel *Helicobacter pylori* CznABC metal efflux pump is required for cadmium, zinc, and nickel resistance, urease modulation, and gastric colonization. Infect Immun.

[44] Dorel C, Lejeune P, Rodrigue A (2006). The Cpx system of *Escherichia coli*, a strategic signaling pathway for confronting adverse conditions and for settling biofilm communities?. Res Microbiol.

[45] Chakraborty S, Winardhi RS, Morgan LK, Yan J, Kenney LJ (2017). Non-canonical activation of OmpR drives acid and osmotic stress responses in single bacterial cells. Nat Commun.

[46] Chen HD, Groisman EA (2013). The biology of the PmrA/PmrB two-component system: The major regulator of lipopolysaccharide modifications. Annu Rev Microbiol.

[47] Lee LJ, Barrett JA, Poole RK (2005). Genome-wide transcriptional response of chemostat-cultured *Escherichia coli* to zinc. J Bacteriol.

[48] Rosenberg H, Gerdes RG, Chegwidden K (1977). Two systems for the uptake of phosphate in *Escherichia coli*. J Bacteriol.

[49] Frawley ER (2018). Nitric oxide disrupts zinc homeostasis in *Salmonella enterica* serovar Typhimurium. MBio.

[50] Rosen DA, Hooton TM, Stamm WE, Humphrey PA, Hultgren SJ (2007). Detection of intracellular bacterial communities in human urinary tract infection. PLoS Med.

[51] Hunstad DA, Justice SS, Hung CS, Lauer SR, Hultgren SJ (2005). Suppression of bladder epithelial cytokine responses by uropathogenic *Escherichia coli*. Infect Immun.

[52] Mulvey MA, Schilling JD, Martinez JJ, Hultgren SJ (2000). Bad bugs and beleaguered bladders: Interplay between uropathogenic *Escherichia coli* and innate host defenses. Proc Natl Acad Sci USA.

[53] Andersen TE (2012). *Escherichia coli* uropathogenesis in vitro: Invasion, cellular escape, and secondary infection analyzed in a human bladder cell infection model. Infect Immun.

[54] Smith SN, Hagan EC, Lane MC, Mobley HL (2010). Dissemination and systemic colonization of uropathogenic *Escherichia coli* in a murine model of bacteremia. MBio.

[55] Merli M (2010). Cirrhotic patients are at risk for health care-associated bacterial infections. Clin Gastroenterol Hepatol.

[56] Bonnel AR, Bunchorntavakul C, Reddy KR (2011). Immune dysfunction and infections in patients with cirrhosis. Clin Gastroenterol Hepatol.

[57] Bunchorntavakul C, Chavalitdhamrong D (2012). Bacterial infections other than spontaneous bacterial peritonitis in cirrhosis. World J Hepatol.

[58] Adams-Sapper S, Diep BA, Perdreau-Remington F, Riley LW (2013). Clonal composition and community clustering of drug-susceptible and -resistant *Escherichia coli* isolates from bloodstream infections. Antimicrob Agents Chemother.

[59] Porcheron G, Garénaux A, Proulx J, Sabri M, Dozois CM (2013). Iron, copper, zinc, and manganese transport and regulation in pathogenic Enterobacteria: Correlations between strains, site of infection and the relative importance of the different metal transport systems for virulence. Front Cell Infect Microbiol.

[60] Subashchandrabose S, Mobley HL (2015). Back to the metal age: Battle for metals at the host-pathogen interface during urinary tract infection. Metallomics.

[61] Subashchandrabose S (2014). Host-specific induction of *Escherichia coli* fitness genes during human urinary tract infection. Proc Natl Acad Sci USA.

[62] Hyre AN, Kavanagh K, Kock ND, Donati GL, Subashchandrabose S (2017). Copper is a host effector mobilized to urine during urinary tract infection to impair bacterial colonization. Infect Immun.

[63] Sabri M, Houle S, Dozois CM (2009). Roles of the extraintestinal pathogenic *Escherichia coli* ZnuACB and ZupT zinc transporters during urinary tract infection. Infect Immun.

[64] Bielecki P (2014). In vivo mRNA profiling of uropathogenic *Escherichia coli* from diverse phylogroups reveals common and group-specific gene expression profiles. MBio.

[65] Wagner D (2005). Elemental analysis of *Mycobacterium avium*-, *Mycobacterium tuberculosis*-, and *Mycobacterium smegmatis*-containing phagosomes indicates pathogen-induced microenvironments within the host cell’s endosomal system. J Immunol.

[66] Subramanian Vignesh K (2016). IL-4 induces metallothionein 3- and SLC30A4-dependent increase in intracellular Zn(2+) that promotes pathogen persistence in macrophages. Cell Rep.

[67] Kitamura H (2006). Toll-like receptor-mediated regulation of zinc homeostasis influences dendritic cell function. Nat Immunol.

[68] Knoell DL (2009). Zinc deficiency increases organ damage and mortality in a murine model of polymicrobial sepsis. Crit Care Med.

[69] Ravasi T, Mavromatis CH, Bokil NJ, Schembri MA, Sweet MJ (2016). Co-transcriptomic analysis by RNA sequencing to simultaneously measure regulated gene expression in host and bacterial pathogen. Methods Mol Biol.

