# Supplementary material for: Uropathogenic Escherichia coli employs both evasion and resistance to subvert innate immune-mediated zinc toxicity for dissemination
Source: Proc Natl Acad Sci U S A. 2019 Mar 7;116(13):6341–50. doi: 10.1073/pnas.1820870116 (PMC6442554; doi:10.1073/pnas.1820870116)
Supplement: Supplementary File [file pnas.1820870116.sapp.pdf]

Supplementary Information for

Uropathogenic *Escherichia coli* employs both evasion and resistance to subvert innate immune-mediated zinc toxicity for dissemination

Claudia J. Stocks[1,2,3], Minh-Duy Phan[3,4], Maud E. S. Achard[3,4], Nguyen Thi Khanh Nhu[3,4], Nicholas D. Condon[1], Jayde A. Gawthorne[3,4], Alvin W. Lo[3,4], Kate M. Peters[3,4], Alastair G. McEwan[3,4], Ronan Kapetanovic[1,2,3,*], Mark A. Schembri[3,4,*] and Matthew J. Sweet[1,2,3,*]

[*]To whom correspondence should be addressed: Prof Matthew Sweet (m.sweet@imb.uq.edu.au), Prof Mark Schembri (m.schembri@uq.edu.au) and Dr. Ronan Kapetanovic (r.kapetanovic@imb.uq.edu.au).

This PDF file includes:

Supplementary text (Materials and methods)
Figs. S1 to S9
Tables S1 to S5
References for citations in SI text

SI Materials and Methods

Generation of zinc reporter plasmids and strains. Oligonucleotides used for the generation of reporter constructs are listed in SI Appendix, Table S4. The GFP chromosomal insert strain was generated using lambda-red recombinase (1). For the GFP plasmid-based reporter, GFP and chloramphenicol resistance cassettes were first amplified and cloned into pQF50 using complementary overhangs. The cloning of the 129 bp *zntA* promoter region upstream of GFP in pQF50 was performed by Epoch Life Science (USA). pQF50-zntA-mCherry was created by PCR amplification of the mCherry fragment, with insertion in place of GFP in pQF50-zntA-GFP via HindIII restriction digestion. The dual reporter plasmid pGcCzntAp, which contains

GFP under the control of the *S.* Typhimurium *rpsM* promoter, as well as mCherry under the control of the *E. coli zntA* promoter, was generated based on the vector pFCcGi (2). DNA manipulation and cloning were performed by Epoch Life Science (USA). Plasmids were transformed into *E. coli* MG1655 or EC958. An overview of plasmids pGcCe and pGcCzntAp is presented in SI Appendix, Fig. S9.

TraDIS. Approximately 2x10^8 cells from a previously constructed miniTn*5* mutant library of EC958 (3) were inoculated into 100 mL of either LB (control) or LB supplemented with 1.0 mM $ZnSO_4$ (test) and incubated for 18 h at 37°C. Subsequently, genomic DNA was extracted from 5 ml of cultures using the Qiagen genomic DNA purification kit. The screening assays were performed in duplicate. TraDIS was performed as previously described (4), using the Nextera DNA library preparation kit (Illumina) with custom primers to specifically amplify and sequence the genomic locations of Tn*5* insertions. All DNA libraries were sequenced on the MiSeq platform at the Queensland Centre for Medical Genomics (Institute for Molecular Bioscience, The University of Queensland). The TraDIS sequence data from this study has been deposited on the Sequence Read Archive (SRA) under the Bio Project number PRJNA416668.

The raw, de-multiplexed fastq files from MiSeq runs were filtered to capture reads containing the 12-bp Tn*5*-specific barcode (5'-TATAAGAGACAG-3'), allowing for 2 mismatches (fastx_barcode_splitter.pl, FASTX-Toolkit v.0.0.13). These reads were trimmed to remove the 12-bp barcode and 58-bp at the 3' end (fastx_trimmer, FASTX-Toolkit v.0.0.13), resulting in high quality sequence reads of 30-bp in length that were mapped to the EC958 chromosome (gb|HG941718) by Maq version 0.7.1 (5). Subsequent analysis steps were carried out using an

in-house Perl script, as previously described (3), to calculate the number of unique insertion sites and the read count at each site for every gene.

Bacterial growth curves. Growth curves were performed in 96-well microtitre plates with 200 µL culture per well at a starting OD_{600} of 0.05. To assess growth in zinc, LB was supplemented with $ZnSO_4$ (Sigma) at 1 mM, unless otherwise indicated. The growth kinetics were monitored every 15 minutes using a FLUOstar OPTIMA plate reader (BMG Labtech) at 37 °C and employing 200 rpm shaking. Growth kinetics in the presence of copper were performed similarly using $CuSO_4$ in complete RPMI (RPMI supplemented with 10% fetal calf serum [Gibco,USA], 2 mM L-glutamine [Gibco], 1 mM sodium pyruvate [Gibco], 10 mM HEPES [Gibco]) or LB. Bacterial cell counts were performed by determining colony forming units at 6 h following growth in the presence of complete RPMI containing 500 µM $CuSO_4$ (the same conditions used to determine the *zntA* mRNA levels reported in Fig. 1*A*). For analysis of combinatorial effects of zinc and reactive oxygen species on bacterial growth, growth curves were performed at 37 °C over a 12 h time course in the presence or absence of paraquat (0.1 mM) and $ZnSO_4$ (0.1 or 0.2 mM). A starting OD_{600} of 0.1 was used, and readings were taken every 20 min, following a 30 s rotation (100 rpm), using a POLARstar Omega spectrophotometer (BMG Labtech).

Quantitative real-time PCR (qRT–PCR). Infected cells and/or bacteria were lysed in TRIzol (Invitrogen) and total RNA was extracted using Direct-zol RNA Miniprep Kit (Zymo), following the manufacturer's instructions. One µg of RNA was treated with Turbo DNA-free (Invitrogen) and then reverse transcribed to cDNA using random hexamers and Superscript III (Life Technologies). Levels of *zntA* mRNA (relative to the control genes *gapA*, *ifhB* or *rrsA*) were quantified by qRT-PCR utilising the Applied Biosystems 7900HT fast RT-PCR system

and the ΔC_t method. Primers used for qRT-PCR (Sigma, Australia) are listed in SI Appendix, Table S5.

Zinc and cathelicidin (LL-37) chequerboard assays. The *in vitro* activity of zinc ($ZnSO_4$) in combination with LL-37 was assessed in a microtitre plate chequerboard assay. The concentration range for $ZnSO_4$ was 0.0625 - 4 mM and for LL-37 was 4 - 64 µg/ml. Assays were performed using $5x10^4$ CFU of each strain in a volume of 100 µl per well; incubation was performed at 37˚C for 18–24 h. The fractional inhibitory concentration (FIC) for each agent was defined as its minimum inhibitory concentration (MIC) in combination divided by its MIC alone. The FIC index (FICI) was the sum of the FIC values for the two agents and was interpreted as previously described: synergy (FICI ≤0.5), additivity (FICI >0.5 to ≤1), no interaction (FICI >1 to ≤4), antagonism (FICI >4).

Flow cytometry. To quantify *zntA*-mediated reporter gene activation by flow cytometry, overnight cultures of *E. coli* were grown in complete RPMI media or LB at 37°C for the indicated time points, after the addition of either water as a control, or zinc sulfate, copper (II) sulfate, ferrous sulfate, magnesium chloride or manganese chloride to a final concentration of 0.5 mM. Following incubation, all tubes were centrifuged and washed twice with PBS, before samples were resuspended in 400 µL of PBS and analysed by flow cytometry using a Gallios Flow Cytometer (Beckman Coulter).

Determination of zinc concentrations by ICP-OES. Bacterial infection assays were performed as described in Experimental Procedures, with HMDM plated overnight in 10 mL media at 5 x 10^6 cells per 10 cm dish (Thermo Fisher Scientific). 'No-cell' controls in the form of 10 mL media on an empty dish were also prepared and left overnight at 37 °C. Cells were

either left uninfected (resting), or infected at MOI 100 with MG1655 or EC958. Prior to infection, bacterial cultures were washed using calcium and magnesium free Hank's balanced salt solution (HBSS, Gibco). At 8 h p.i., cells were washed twice with HBSS, lysed in 5 mL of lysis solution (0.1% sodium dodecyl sulfate in MilliQ water), and placed into treated, pre-weighed 10 mL plastic tubes (School of Earth and Environmental Sciences, University of Queensland, Australia). Triple distilled HNO_3 (School of Earth and Environmental Sciences) was then added to acidify the sample to 2%. The sample was then made up to a total volume of 10 mL with MilliQ water. Samples were analysed using an Optima 8300 DV ICP-OES spectrometer (Perkin Elmer, USA). Freshly prepared calibration standards were used to estimate intramacrophage zinc concentrations (with two or three spectral lines measured as a quality control). Values obtained in the 'No-cell' controls were subtracted from the values obtained from the macrophage lysates. The overall concentration of zinc within a macrophage was approximated on the basis of the zinc concentration within 1 million macrophages lysed in 1 mL of lysis buffer (as determined by ICP-OES), the approximate volume of an alveolar macrophage (4990 μm^3) calculated by Krombach et al (6), and the atomic mass of Zn (65.38).

SI Figures

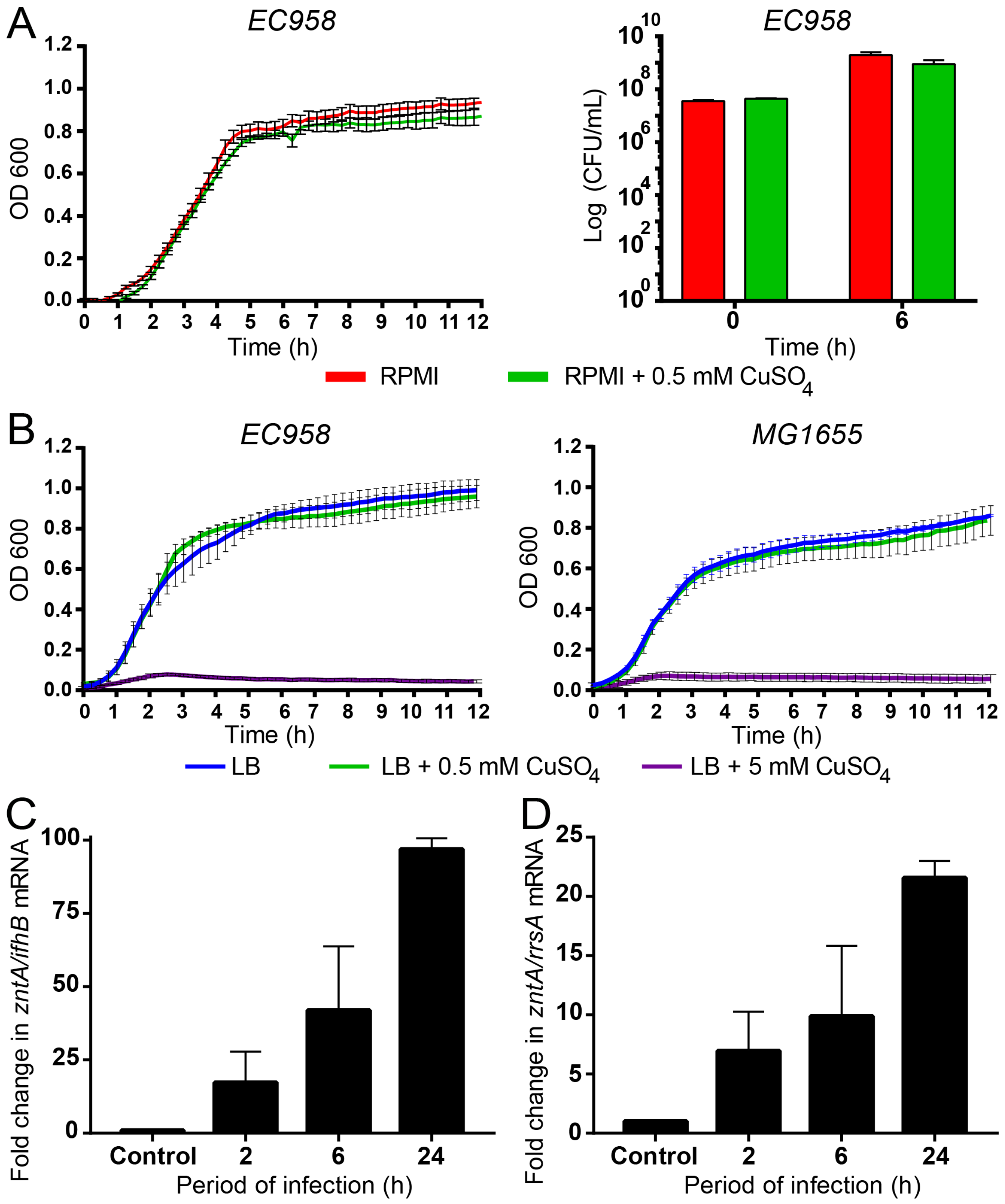

Fig. S1. Controls for regulation of *zntA*. (A) Growth kinetics and CFU data for EC958 grown in complete RPMI ± 0.5 mM $CuSO_4$. OD 600 was recorded using a FLUOstar OPTIMA plate reader over a 12 h period. CFU counts were determined at the start of the experiment and after 6 h incubation (the same conditions used to determine the *zntA* mRNA levels reported in Fig. 1*A*). Data (n=3, mean ± SEM) are combined from 3 experiments. **(B)** Growth kinetics of EC958 and MG1655 in LB containing the indicated concentrations of $CuSO_4$, with OD 600 recorded for 12 h as above. Data (n=3, mean ± SEM) are combined from 3 experiments. **(C-D)** HMDM were infected with EC958 (MOI 100) for 1 h, whereupon the media was removed and extracellular bacteria removed by gentamicin exclusion. Cells were lysed at 2, 6 or 24 h post-infection, after which RNA was extracted. Levels of *zntA* mRNA, relative to either *ifhB* **(C)** or *rrsA* **(D)**, were determined by qPCR. Bacteria cultured for 2 h in complete RPMI media alone served as a control. Data (mean + range, n=2) are combined from 2 independent experiments, and are displayed as fold change relative to the control.

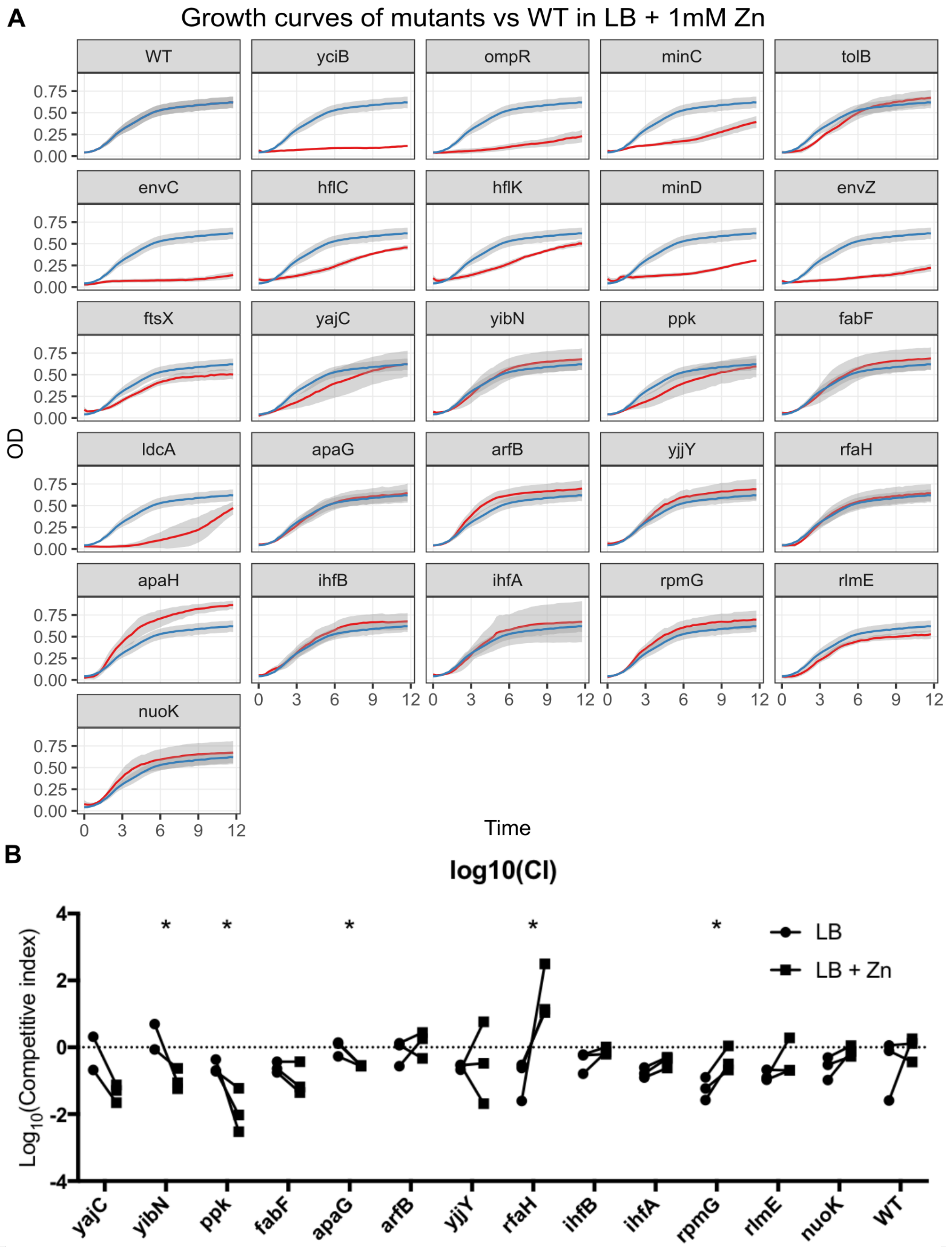

Fig. S2. Growth curves and competition assays with mutant strains for genes identified by TraDIS. (A) Growth kinetics of wild-type (blue line) versus mutants (red line) in LB-$ZnSO_4$ conditions. Data (n=3) are combined from 3 independent experiments, presented as mean (solid line) ± SEM (grey shades). **(B)** Competition assays were performed in triplicate in 96-well plates with the mutant of interest mixed in a 1:1 ratio with EC958 Δlac reference strain at a starting OD of 0.05. Each competitive pair was grown in LB and LB supplemented with 1 mM zinc, at 37°C and 200 rpm. Viable counts were performed at 0 and 18 h by serial dilution and plating on MacConkey agar. Competitive Index (CI) of each mutant compared to wild-type EC958 was calculated using the formula CI = $(CFU_{mutant\ at\ t18}/CFU_{mutant\ at\ t0})$ / $(CFU_{EC958\Delta lac\ at\ t18}/CFU_{EC958\Delta lac\ at\ t0})$, and Student's t-tests were performed to determine any significant fitness difference. * denotes p<0.05.

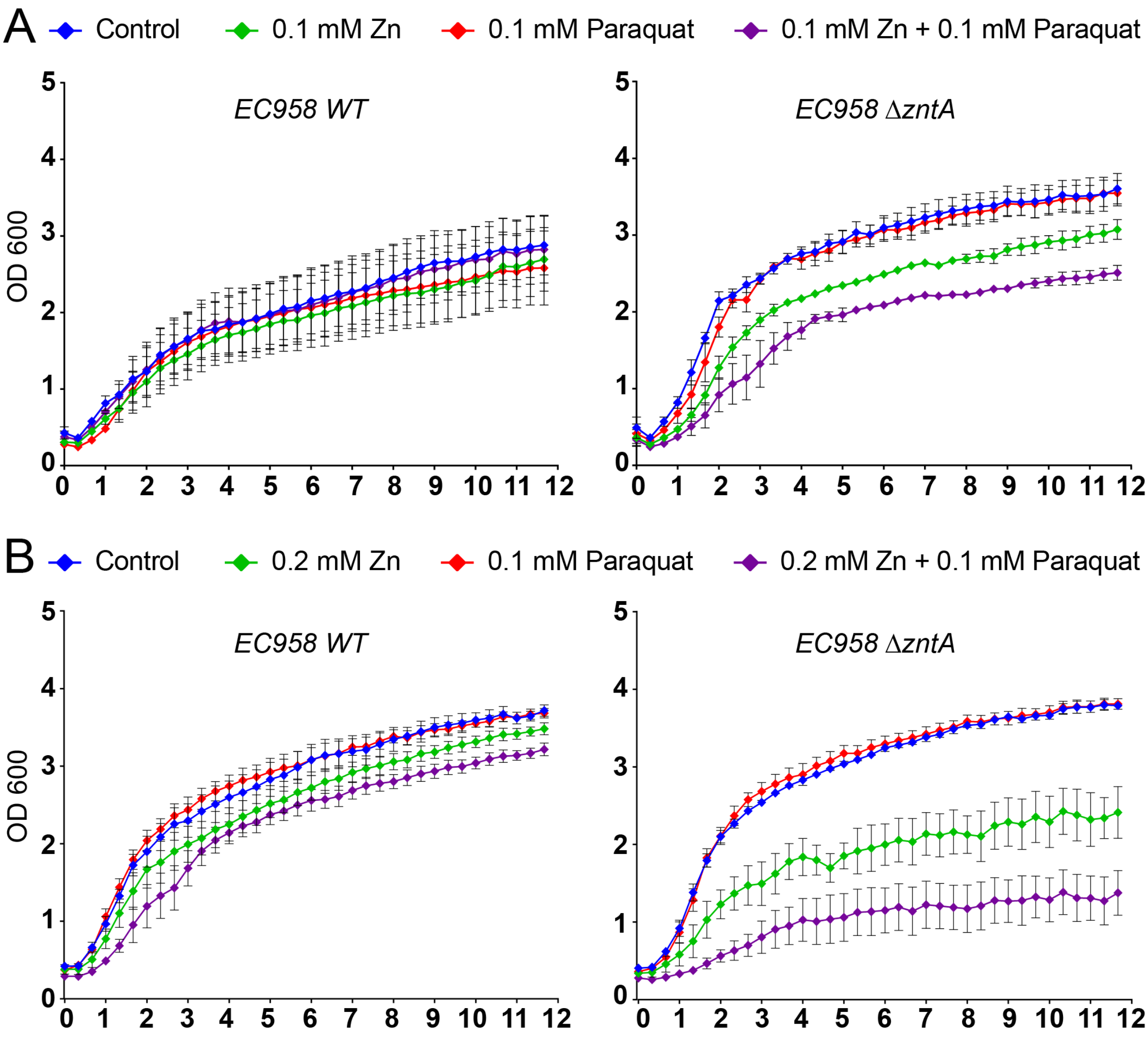

Fig. S3. Paraquat enhances the inhibitory effects of zinc on growth of an EC958 *zntA* mutant strain. Overnight bacterial cultures of wild-type or *zntA* mutant EC958 were diluted to an OD 600 of 0.1 then grown at 37˚C for 12 h using a POLARstar Omega spectrophotometer, with shaking and OD measurements every 20 min. Cultures were supplemented with water (control) or 0.1 mM paraquat in the presence or absence of **(A)** 0.1 mM $ZnSO_4$ or **(B)** 0.2 mM $ZnSO_4$. Data are combined from **(A)** (n=3, ± SEM) or **(B)** (n=4, ± SEM) independent experiments, and are presented as mean (solid line).

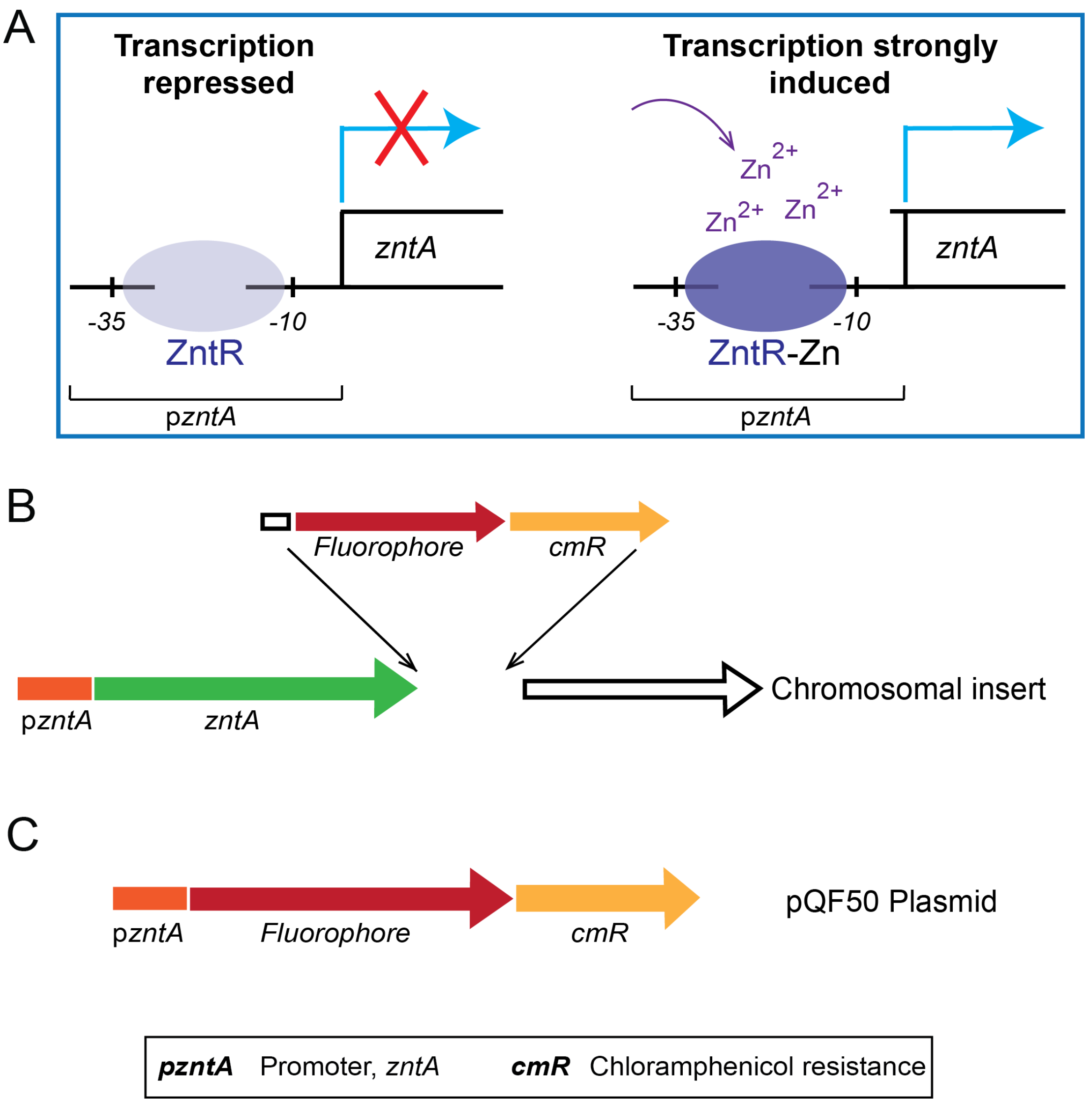

Fig. S4. Workflow of *zntA* reporter design. (A) In *E. coli*, the transcription of *zntA* is controlled by ZntR, which binds and represses the promoter in the absence of zinc. Upon binding zinc, ZntR is converted into a transcriptional activator and activates the *zntA* promoter. As shown in **(B)**, a chromosomal zinc-reporter strain was generated whereby the sequence for a fluorophore and chloramphenicol resistance was inserted directly downstream of zntA. **(C)** Depicts the sequence that was inserted into the pQF50 plasmid, being the promotor of *zntA*, fluorophore and chloramphenicol resistance. For both **(B)** and **(C)**, increasing zinc concentrations induce the expression of *zntA*, and also result in the co-expression of a fluorophore that can be detected by flow cytometry or fluorescence microscopy.

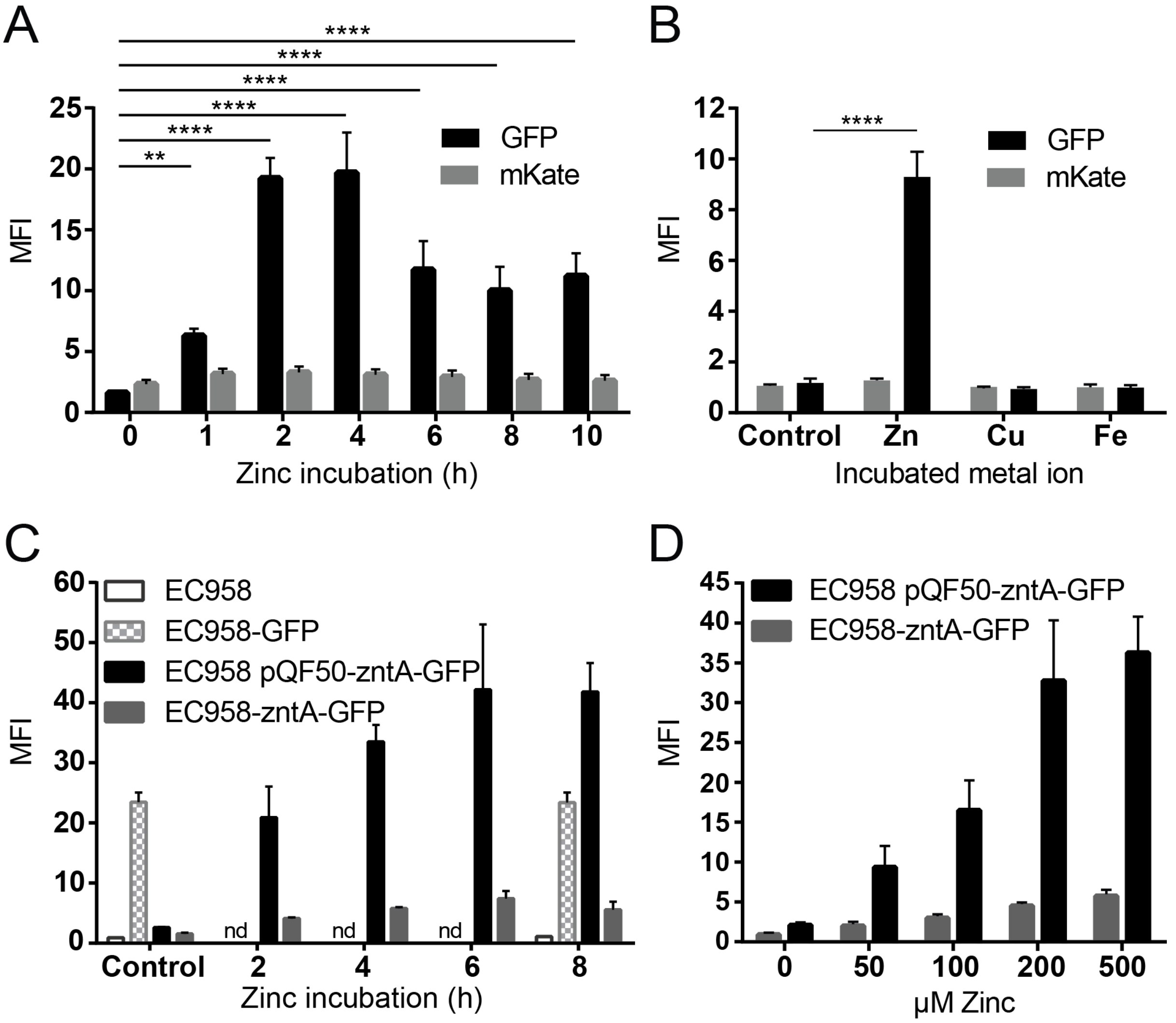

Fig. S5. Generation and testing of chromosomal insert and plasmid-based GFP zinc-stress reporter strains. (A) An EC958 strain constitutively expressing mKate and containing the *gfp* gene inserted downstream of *zntA* on the chromosome (EC958-zntA-GFP) was incubated in complete RPMI media containing 500 μM $ZnSO_4$ over an 8 h time course, before median GFP and mKate fluorescence was determined by flow cytometry. Data (mean + SEM, n=3) are combined from 3 independent experiments. **(B)** EC958-zntA-GFP was incubated in complete RPMI media containing 500 μM $ZnSO_4$, $CuSO_4$, or $FeSO_4$, with water used as a control. Data (mean + SEM, n=3) are combined from 3 independent experiments. For both **A-B**, statistical analysis was performed using a two-way ANOVA with Sidaks's multiple comparisons test, comparing the mean fluorescence of each time point with the control mean (** denotes $p<0.01$, **** $p<0.0001$, and all other comparisons were not significant). **(C)** Wild-type EC958, EC958-GFP (constitutively expresses GFP) and the two zinc stress reporter strains EC958 pQF50-zntA-GFP (plasmid-encoded zntA-GFP reporter) and EC958-zntA-GFP (chromosomal zntA-GFP reporter) were incubated in LB plus 500 μM zinc sulfate over an 8 h time-course, before GFP fluorescence was assessed by flow cytometry. In **(D)**, EC958-zntA-GFP and EC958 pQF50-zntA-GFP were incubated in complete RPMI media containing increasing concentrations of zinc sulfate (0 to 500 μM), before being assessed by flow cytometry. For both **C-D**, data are combined from 2 independent experiments (n=2, mean + range). nd = not determined.

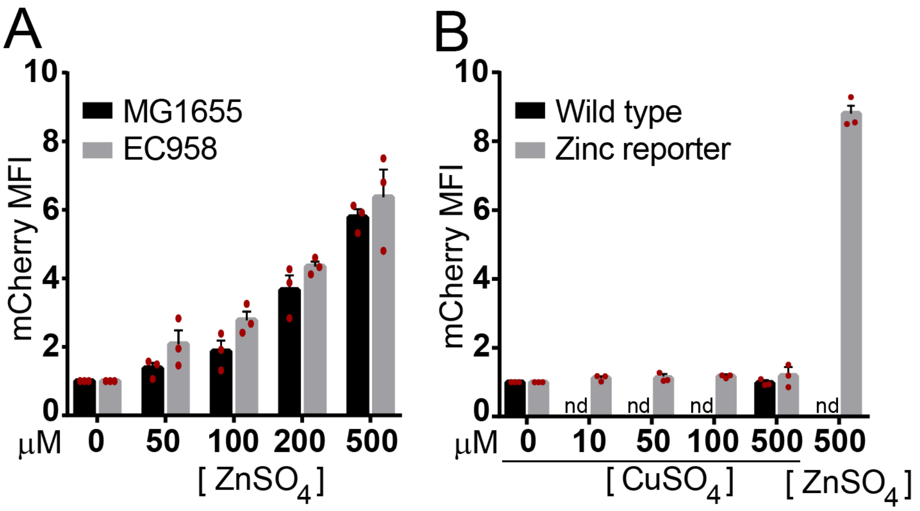

Fig. S6. Sensitivity and specificity controls for MG1655 and EC958 zinc-reporter strains. (A) MG1655-pQF50-zntA-mCherry and EC958-pQF50-zntA-mCherry were cultured in complete RPMI media supplemented with 50, 100, 500 or 1000 μM $ZnSO_4$, or a no zinc control for 8 h, after which median mCherry fluorescence was determined by flow cytometry. Data (mean + SEM, n=3) are combined from 3 independent experiments, and represent fold change compared to the no zinc control. **(B)** MG1655 (Wild-type) or MG1655-pQF50-zntA-mCherry (Zinc reporter) were cultured as above with 0, 10, 50, 100, 200, 500 μM $CuSO_4$ or 500 μM $ZnSO_4$ (positive control) for 4 h. Median mCherry fluorescence was determined by flow cytometry. Data (mean + SEM, n=3) are combined from 3 independent experiments, and represent fold change compared to the no copper control. nd = not determined.

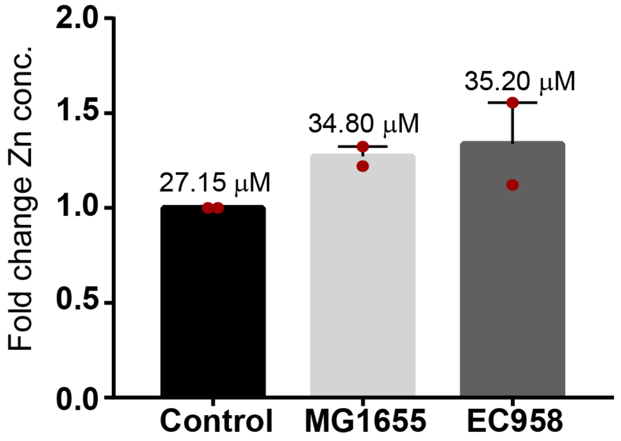

Fig. S7. Zinc concentrations within control and *E. coli*-infected HMDM. HMDM were plated at a density of 5 x 10^6 cells/10 mL on a 10 cm dish overnight, after which they were infected with MG1655 or EC958 (MOI 100) or left uninfected as a control. At 1 h p.i., gentamicin exclusion was performed, and at 8 h p.i., cells were washed twice with HBSS, before being lysed in 5 mL lysis buffer (0.1% SDS in MilliQ water). Zinc concentrations were determined by ICP-OES, and values obtained for media in an empty dish that had been treated identically were subtracted from the experimental samples. Data (mean + range, n=2) are combined from 2 independent experiments (each experiment contained 2 replicates) and are presented as fold change relative to the uninfected control cells. The values above each bar represent the estimated mean intramacrophage zinc concentration from the 2 experiments.

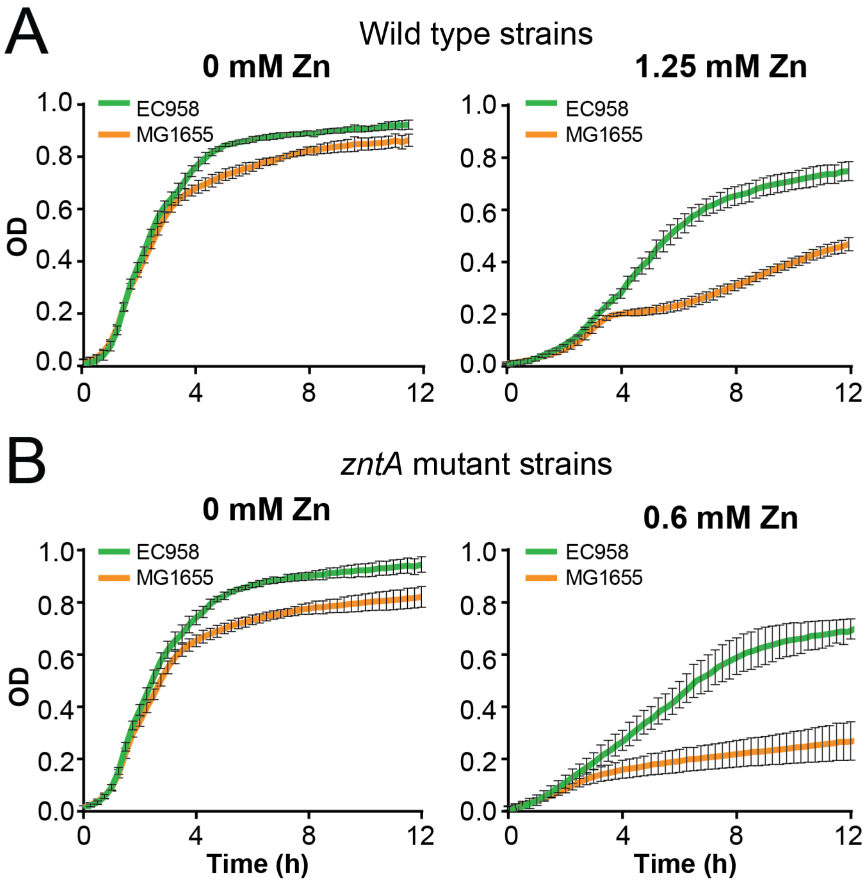

Fig. S8. Wild type EC958 and an EC958 *zntA* mutant are more resistant to zinc than their MG1655 equivalents. Growth kinetics of EC958 (green line) versus MG165 (orange) was determined for **(A)** wild type and **(B)** *zntA* mutant strains. Bacteria were grown for 12 h in a 96-well microtitre plate in LB supplemented with the indicated zinc sulfate concentrations at 37°C with 200 rpm shaking and OD read in a FLUOstar OPTIMA. Data (mean ± SEM, n=3) are combined from 3 independent experiments.

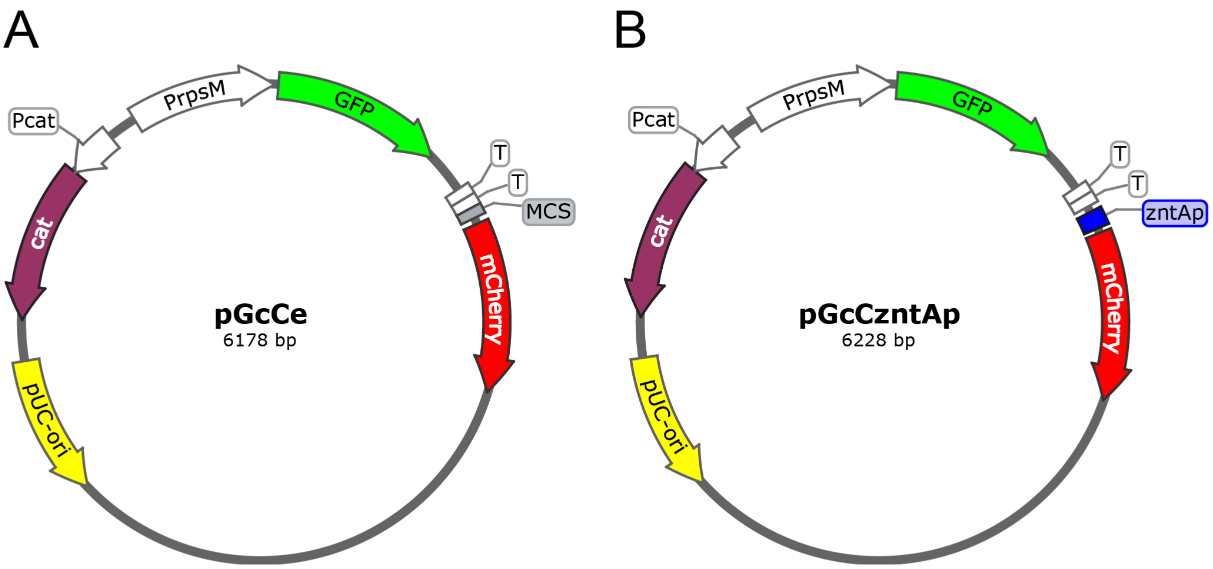

Fig. S9. Plasmid maps of dual reporter vectors used in this study. (A) Map of pGcCe showing the position of pUC origin of replication (yellow), chloramphenicol gene cassette (*cat* gene and Pcat promoter), *gfp* gene under PrpsM promoter (green) with two terminators T at its 3'-end and *mCherry* gene (red) with a multiple cloning site (MCS) upstream for cloning of promoter of interest. **(B)** Map of pGcCzntAp showing the vector pGcCe with zntAp promoter (blue) inserted at the MCS. The *in silico* design and cloning were performed using CLC Main Workbench, and the Figure was prepared with SnapGene Viewer. To generate these plasmids, first the *bla* gene in the pFCcGi vector was swapped for the *cat* gene to enable Cm selection in strains derived from EC958; then the location of GFP and mCherry in pFCcGi were swapped. The *araC*-pBAD fragment in pFCcGi was also replaced with terminators and a multiple cloning site, resulting in the vector pGcCe (**A**). The promoter region of *zntA* (zntAp) was then cloned into pGcCe to generate pGcCzntAp (**B**).

SI Tables

Table S1. TraDIS-identified genes that regulate growth in the presence of Zn

EC958 locus tag	Gene Name	MG1655 locus tag	Product	logFC	Growth effects in $ZnSO_4$
EC958_4198	***pstB***	b3725	phosphate transporter subunit	-7.81	Decrease[a]
EC958_4397	***cpxR***	b3912	DNA-binding response regulator in two-component regulatory system with CpxA	-6.09	Decrease[a]
EC958_3799	*ompR*	b3405	DNA-binding response regulator in two-component regulatory system with EnvZ	-5.24	Decrease[a]
EC958_1529	*yciB*	b1254	predicted inner membrane protein	-5.16	Decrease[a]
EC958_4662	*hflC*	b4175	modulator for HflB protease specific for phage lambda cII repressor	-4.99	Decrease[a]
EC958_4661	*hflK*	b4174	modulator for HflB protease specific for phage lambda cII repressor	-4.86	Decrease[a]
EC958_1414	*minC*	b1176	cell division inhibitor	-4.28	Decrease[a]
EC958_0859	*tolB*	b0740	periplasmic protein	-4.12	Decrease[a]
EC958_3863	***zntA***	b3469	zinc, cobalt and lead efflux system	-3.93	Decrease[a]
EC958_3856	*ftsX*	b3462	predicted transporter subunit: membrane component of ABC superfamily	-3.79	Decrease[a]
EC958_1413	*minD*	b1175	membrane ATPase of the MinCDE system	-3.78	Decrease[a]
EC958_4021	*envC*	b3613	activator of AmiB,C murein hydrolases, septal ring factor	-3.70	Decrease[a]
EC958_1431	*ldcA*	b1192	murein tetrapeptide carboxypeptidase; LD-carboxypeptidase A	-3.60	Decrease[a]
EC958_4019	*yibN*	b3611	predicted rhodanese-related sulfurtransferase	-3.32	Decrease[b]
EC958_1291	*fabF*	b1095	3-oxoacyl-[acyl-carrier-protein] synthase II	-3.32	No change[c]
EC958_0545	*yajC*	b0407	SecYEG protein translocase auxillary subunit	-3.32	No change[c]
EC958_3798	*envZ*	b3404	sensory histidine kinase in two-component regulatory system with OmpR	-3.19	Decrease[a]
EC958_3686	***zntR***	b3292	DNA-binding transcriptional activator in response to Zn(II)	-3.11	Decrease[a]
EC958_2807	*ppk*	b2501	polyphosphate kinase, component of RNA degradosome	-3.09	Decrease[b]
EC958_0133	*yjjY*	b4402	predicted protein	4.08	No change[c]
EC958_3579	*rlmE*	b3179	23S rRNA U2552 2'-O-ribose methyltransferase, SAM-dependent	4.14	No change[c]
EC958_1934	*ihfA*	b1712	integration host factor (IHF), DNA-binding protein, alpha subunit	4.14	No change[c]
EC958_2614	*nuoK*	b2279	NADH:ubiquinone oxidoreductase, membrane subunit K	4.27	No change[c]
EC958_4044	*rpmG*	b3636	50S ribosomal subunit protein L33	4.32	Increase[b]
EC958_0186	*apaG*	b0050	protein associated with Co2+ and Mg2+ efflux	4.39	Increase[b]
EC958_0338	*arfB*	b0191	alternative stalled-ribosome rescue factor B; peptidyl-tRNA hydrolase, ribosome-attached	4.75	No change[c]
EC958_4322	*rfaH*	b3842	DNA-binding transcriptional antiterminator	4.92	Increase[b]
EC958_1122	*ihfB*	b0912	integration host factor (IHF), DNA-binding protein, beta subunit	4.96	No change[c]
EC958_0185	*apaH*	b0049	diadenosine tetraphosphatase	5.17	Increase[a]
EC958_3889	*pitA*	b3493	Zinc/phosphate transporter, low-affinity	5.91	Increase[a]

[a] Growth effects were determined by comparing growth kinetics of the wild-type and targeted mutants of the genes of interest in LB supplemented with 1 mM $ZnSO_4$.
[b] Growth effects were determined by competitive growth assays between wild-type and targeted mutants in LB versus LB supplemented with 1 mM $ZnSO_4$. These mutants did not exhibit any change in single growth experiments in LB supplemented with 1 mM $ZnSO_4$.
[c] No growth effect in single or competitive assays in LB supplemented with 1 mM $ZnSO_4$.
Mutants of the genes highlighted in bold were examined in intramacrophage infection assays.

Table S2. Summary of microtitre plate chequerboard assay examining synergy between cathelicidin (LL-37) and $ZnSO_4$ against wild type EC958 and EC958*ΔzntA*

Strain	No. of replicates	Average FICI	SD	Nature of effect*
EC958	5	2.96	0.50299105	indifference
EC958 *ΔzntA*	2	2	0	indifference

Fractional inhibitory concentration index (FICI). Calculated and interpreted as per F.C Odds, 2003 (7).
*Interaction: FICI ≤0.5, synergism; 0.5 < FICI < 4, indifference.

Table S3. Strains and plasmids used in this study

**Name**	**Description**	**Reference**
MG1655	*E. coli* K-12 strain MG1655	(8)
EC958	ST131 reference strain EC958	(9)
EC958-zntA-GFP	EC958 with GFP inserted directly downstream of *zntA*	This study
pQF50	Empty vector	(10)
pQF50-zntA-GFP	Promoter of *zntA*, GFP and chloramphenicol cassette cloned into pQF50	This study
pQF50-zntA-mCherry	mCherry cloned into pQF50-zntA-GFP in place of GFP	This study
pGcCe	Plasmid with constitutive GFP fluorescence driven by the *S.* Typhimurium *rpsM* promoter; mCherry present with no promoter to drive expression	This study
pGcCzntAp	Promoter of *zntA* cloned into pGcCe to drive zinc-inducible mCherry expression	This study

Table S4. List of primers used for generation of reporter plasmids, reporter strains and mutants

**Primer number/name**	**Sequence (5'-3')**
RBS-GFP-F	GGATCCTCTAGATTTAAGAAGGAG
GFP-Cm-R	TATGCCGGTGTCTCTTAGTC
EC958_tag_zntA_Fwsc	GCTGCCGGAAAACATCCT
EC958_tag_zntA_Fwup	GGGTGTCATTGCGTTGCAG
EC958_tag_zntA_Rvup	CTCCTTCTTAAATCTAGAGGATCCTCAATGTTGCGATCGGTTTG
EC958_tag_zntA_Fwdn	GACTAAGAGACACCGGCATATGGGGAGAGGGTTAGGGTGA
EC958_tag_zntA_Rvdn	TATAGCGTTGCGTTTGCGAG
EC958_tag_zntA_Rvsc	ATGGAGACCGGAAGCTGAAC
pQF50.invR	CTAGCTAGAAGCTTCTAGAGATCC
pQF50.invF	GACTAAGAGACACCGGCATATGGAGTGCGATCTTCCTGAG
RBS-GFP-F2	TTTAAGAAGGAGATATACATATGAGTAAAGGAGAAGAACTTTTC
XhoI-zntAp-F	ACTTCCCTCGAGTCCGCTCGCTGTATCTCTG
BamHI-zntAp-R	CAAATTGGATCCCTTTCTTGCCGTGATTGTC
HindIII_CmFW	GACTAGAAGCTTGTTCCTATTCCGAAGTTCC
CmRV_EcoRV	TCAGAGATATC TATGCCGGTGTCTCTTAGTC
HindIII_mCherry_FW	TGATCAAGCTTCGCGTTAACTTCGATCTAC
mCherry_RV	GGTGCCGAGGATGACGATGA
pQF50_screen_FW	AGGAAGCAGCCCAGTAGTAG
mCherry_screen_RV	GAACTGAGGGGACAGGATGT
7963-MGzntA-Fwsc	CTCGCCACTAAACTGGAAGC
5063-MGzntA-Fwup	CGTCAACGTCTGATGTACGC
5064-MGzntA-Rvup	GGACCATGGCTAATTCCCATCAGGAGTCGACATGGCATC
5065-MGzntA-Fwdn	CGAAGCAGCTCCAGCCTACACTGTTGCGCAGGAAATAAGG

5066-MGzntA-Rvdn	CGCCGCATGATACTGCTT
5067-MGzntA-Rvsc	GGTGAGGGAACCCAATACG
7964-MGzntR-Fwup	CCGTAGTATCCCCACTTATC
7965-MGzntR-Rvup	GGAATAGGAACTAAGGAGGATACTTCCGCCATTTTGCCA
7966-MGzntR-Fwdn	CCTACACAATCGCTCAAGACCTGCTCATAGCAGTGTTTATTG
7967-MGzntR-Rvdn	GAATCGGAAAAAGAAGAACCAG
6147_MGzntR_Fwsc	CACTCGTATTCTGAAGTGTGG
6148_MGzntR_Rvsc	ATCCCTGCTGTTGTGATGC
8909_MGcpxR_Fwup	CATCTCAACCTGACGAGCAA
8910_MGcpxR_Rvup	GGAATAGGAACTAAGGAGGAATCAACTAACAGGATTTTATTCATTGT
8911_MGcpxR_Fwdn	CCTACACAATCGCTCAAGACAGATGGTCACCCGTGGTTTA
8912_MGcpxR_Rvdn	TTGGTAATTATCTTCGCCATCA
5713_MGcpxR-Fwsc	ACTGCTCCATTCGTTGTTGA
5714_MGcpxR-Rvsc	GGGCGGTCAAACAGTAAGTT
8905_MGpstB_Fwup	AGATGATCTCTGCGATTACGC
8906_MGpstB_Rvup	GGAATAGGAACTAAGGAGGATTTACTCGGGGCAGTTTCA
8907_MGpstB_Fwdn	CCTACACAATCGCTCAAGACCTGGGCGAATTGATTGAGTT
8908_MGpstB_Rvdn	CCAGCGACTCCAGACTTACC
5953_MGpstB_Fwsc	CGTCGATTGTGGTTGGTC
5954_MGpstB_Rvsc	TACCGCTTCGTCAATGTCC

Table S5. List of primers used for qPCR

Primer name	Sequence (5'-3')	Product size
*E.coli*_ZntA_Fw<br>*E.coli*_ZntA_Rv	CGAAGCACAGGTTGCTGAAC<br>CGCAAATCAATACGCGCTCA	107 bp
*E.coli*_GAPA_Fw<br>*E.coli*_GAPA_Rv	GGTGCGAAGAAAGTGGTTATGAC<br>GGCCAGCATATTTGTCGAAGTTAG	88 bp
*E.coli*_ifhB_Fw<br>*E.coli*_ifhB_Rv	GCGGTTTCGGCAGTTTCT<br>CGCAGTTCTTTACCAGGTTT	124 bp
*E.coli*_rrsA_Fw<br>*E.coli*_rrsA_Rv	CTCTTGCCATCGGATGTGCCCA<br>GTGTGGCTGGTCATCCTCTCA	102 bp

SI References

1. Datsenko KA & Wanner BL (2000) One-step inactivation of chromosomal genes in Escherichia coli K-12 using PCR products. *Proc Natl Acad Sci USA* 97(12):6640-6645.
2. Figueira R, Watson KG, Holden DW, & Helaine S (2013) Identification of salmonella pathogenicity island-2 type III secretion system effectors involved in intramacrophage replication of S. enterica serovar typhimurium: implications for rational vaccine design. *mBio* 4(2):e00065.
3. Phan MD*, et al.* (2013) The serum resistome of a globally disseminated multidrug resistant uropathogenic Escherichia coli clone. *PLoS Genet* 9(10):e1003834.
4. Hancock SJ*, et al.* (2017) Identification of IncA/C Plasmid Replication and Maintenance Genes and Development of a Plasmid Multilocus Sequence Typing Scheme. *Antimicrob Agents Chemother* 61(2):pii: e01740-01716.
5. Li H, Ruan J, & Durbin R (2008) Mapping short DNA sequencing reads and calling variants using mapping quality scores. *Genome Res* 18(11):1851-1858.
6. Krombach F, et al. (1997) Cell size of alveolar macrophages: an interspecies comparison. *Environ Health Perspect* 105:1261-1263.
7. Odds FC. (2003) Synergy, antagonism, and what the chequerboard puts between them. *J Antimicrob Chemother* 52:1.

8. Blattner FR, et al. (1997) The complete genome sequence of Escherichia coli K-12. *Science* 277:1453-62.
9. Totsika M, et al. (2011) Insights into a multidrug resistant Escherichia coli pathogen of the globally disseminated ST131 lineage: genome analysis and virulence mechanisms. *PLoS One* 6:e26578.
10. Farinha MA, Kropinski AM. (1990) Construction of broad-host-range plasmid vectors for easy visible selection and analysis of promoters. *J. Bacteriol.* 172:3496-9.
